# Interpreting ancient food practices: stable isotope and molecular analyses of visible and absorbed residues from a year-long cooking experiment

**DOI:** 10.1038/s41598-020-70109-8

**Published:** 2020-08-27

**Authors:** Melanie J. Miller, Helen L. Whelton, Jillian A. Swift, Sophia Maline, Simon Hammann, Lucy J. E. Cramp, Alexandra McCleary, Geoffrey Taylor, Kirsten Vacca, Fanya Becks, Richard P. Evershed, Christine A. Hastorf

**Affiliations:** 1 Department of Anatomy, University of Otago, Dunedin, New Zealand; 2 Archaeological Research Facility, University of California, Berkeley, USA; 3 Organic Geochemistry Unit, School of Chemistry, University of Bristol, Bristol, UK; 4 Bernice Pauahi Bishop Museum, Honolulu, HI USA; 5 Gould School of Law, University of Southern California, Los Angeles, USA; 6 Department of Anthropology and Archaeology, School of Arts, University of Bristol, Bristol, UK; 7 Department of Chemistry and Pharmacy, Friedrich-Alexander University Erlangen-Nürnberg, Erlangen, Germany; 8 Department of Anthropology, University of California, Berkeley, USA; 9 Social Sciences Division, University of Hawai’i - West O’ahu, Kapolei, HI USA; 10 Unaffiliated, Santa Barbara, CA USA

**Keywords:** Biogeochemistry, Environmental sciences

## Abstract

Chemical analyses of carbonized and absorbed organic residues from archaeological ceramic cooking vessels can provide a unique window into the culinary cultures of ancient people, resource use, and environmental effects by identifying ingredients used in ancient meals. However, it remains uncertain whether recovered organic residues represent only the final foodstuffs prepared or are the accumulation of various cooking events within the same vessel. To assess this, we cooked seven mixtures of C_3_ and C_4_ foodstuffs in unglazed pots once per week for one year, then changed recipes between pots for the final cooking events. We conducted bulk stable-isotope analysis and lipid residue analysis on the charred food macro-remains, carbonized thin layer organic patina residues and absorbed lipids over the course of the experiment. Our results indicate that: (1) the composition of charred macro-remains represent the final foodstuffs cooked within vessels, (2) thin-layer patina residues represent a mixture of previous cooking events with bias towards the final product(s) cooked in the pot, and (3) absorbed lipid residues are developed over a number of cooking events and are replaced slowly over time, with little evidence of the final recipe ingredients.

## Introduction

The study of ancient cooking pots resonates with a fundamental aspect of what it means to be human—the transformation of raw ingredients into a meal is a shared human experience throughout history^1–3^. Meals are representations of the (paleo)environment, revealing the resources that are harvested from local areas or shedding light on exchange networks^1,4^. The foods consumed can also represent the values of the people who eat them^5–7^, being often a smaller selection of what their environment has to offer, and reflecting specific culturally-based choices about what constitutes any item as “food”^8^. Analysis of the residues from cooking pots and other materials (for example, lithic tools) can bring us one step closer to reconstructing the environmental and social circumstances of our ancestors^1^.

Culturally-driven food preferences and practices are often difficult to discern from archaeological datasets: while fragments of past cooking activities and meals may be recovered through archaeobotanical and zooarchaeological evidence, rarely are these lines of evidence sufficient to capture the entire range of plant and animal species that were prepared for consumption, or their relative importance (and therefore, degree of exploitation) within a particular society and ecosystem context. Cooking practices can incorporate a diverse array of materials (ceramic vessels, basketry, leaves, wooden dishes, sticks/skewers of (in)organic materials, hot stones, lithic tools, and more) and preparation techniques (boiling, roasting, braising, baking, fermenting, etc.). Pioneering studies beginning in the 1970s demonstrated that organic residues recovered from archaeological materials retained aspects of their chemical composition^9–14^. Chemical analyses of lipids absorbed into the fabric of unglazed pottery vessels (the primary residue form studied in archaeology), in conjunction with carbonized surface residues, including charred food macro-remains and deposited thin-layer patina residues, are lines of evidence towards understanding ancient culinary practices by potentially providing a direct reflection of the original contents of the ceramic vessels^14–21^. Carbonized food residues derived from the burning of food during cooking have been hypothesised to represent a snapshot of the foodstuffs cooked within the vessel. However, it is not certain whether these organic residues reflect only the most recent ingredients prepared in the vessel or if they are the accumulation of cooking events across the vessel’s use life. It is important to understand the relationship between the use of the vessel over its lifespan, and the residues ultimately extracted and analyzed.

Such residues have been investigated using bulk carbon and nitrogen stable isotope approaches, with ingredient compositions determined using the principles applied in food web studies^11,14,22–24^. This method allows broad assignment of the contributions of C_3_ and C_4_ plants, terrestrial, and aquatic foodstuffs to be assessed in these carbonized remains. Similarly, lipid residue analysis can be performed on both carbonized and absorbed residues^16,25^, which provides biomarker evidence for the natural products processed by people in the past^20,26^. The range of food-related commodities detectable in pottery vessels continues to increase, and currently includes: terrestrial animal products^27,28^, leafy vegetables^13,29^, specific plant oils^30,31^, beeswax^32^^,^ cereals^33^ and aquatic commodities^18,25,34^.

Several studies have shown a correspondence between the bulk *δ*^13^C values of carbonized residues and absorbed lipids, where the combination of analyses have provided complementary data to support conclusions regarding vessel use^35–40^. For example, both bulk *δ*^13^C values and individual fatty acid *δ*^13^C values can discriminate between habitats, such as terrestrial versus aquatic species (and further refinement between freshwater, marine, and anadromous species), since marine species are ^13^C enriched^35–39,41^.

Comparisons of lipid and bulk residue compositions can vary due to differential preservation of lipids and other food constituents in different sample types^42,43^. Cooking methods and residue formation processes will also differentially impact the degree to which analyzed carbon and nitrogen reflect contributions from carbohydrates, lipids, and proteins. Equally, isotopic differences will exist between superficial residues composed of carbonized products (created by Maillard reactions and therefore predominantly carbohydrate and protein based) and absorbed residues (predominantly lipid based) due to the differences in composition of these residue types^28^. Differences in the inferred compositions of carbonized and absorbed lipid residues have also been hypothesised to represent different periods of vessel use (i.e. multiple uses versus the final use)^42^. As noted in Roffet-Salque et al.^4^ “…the majority of analyses now target absorbed organic residues in unglazed ceramic vessels, which generally originate from the original contents that were either stored or processed in the vessels, representing either a single use or an accumulation of cooking events over a vessel’s lifetime” (Roffet-Salque et al., p. 627). However, there is currently limited data on lipid turnover as it has been assumed that lipids are absorbed over the lifetime of the pot, and little is known about turnover speed and timing in the ceramic matrix once lipids have been incorporated^44^. Our study aims to address how a pot’s cooking life history shapes its residues and the resulting chemical fidelity of the residues in comparison to the original ingredients^44–47^.

Here we report the results of a year-long cooking experiment (minimum of 52 cooking episodes per pot) designed to test the following research questions:

(i) Do surface residues (food crust macro-remains and thin-layer organic patina deposits) reflect a single cooking event, specifically the final use, or are surface residues a mixture of previous cooking events in that vessel?
(ii) Do the stable isotopic values of macroscopic carbonized residues relate to the biogenic isotopic values of the original (first) ingredients cooked in the pot or the final ingredients prepared in the pot?
(iii) Are absorbed lipid residues accumulated over the use lifetime of the vessel?
(iv) Do absorbed lipid residues represent the early cooking history, becoming saturated after multiple cooking events, or indicate the final cooking event(s)?

**Figure 1:**
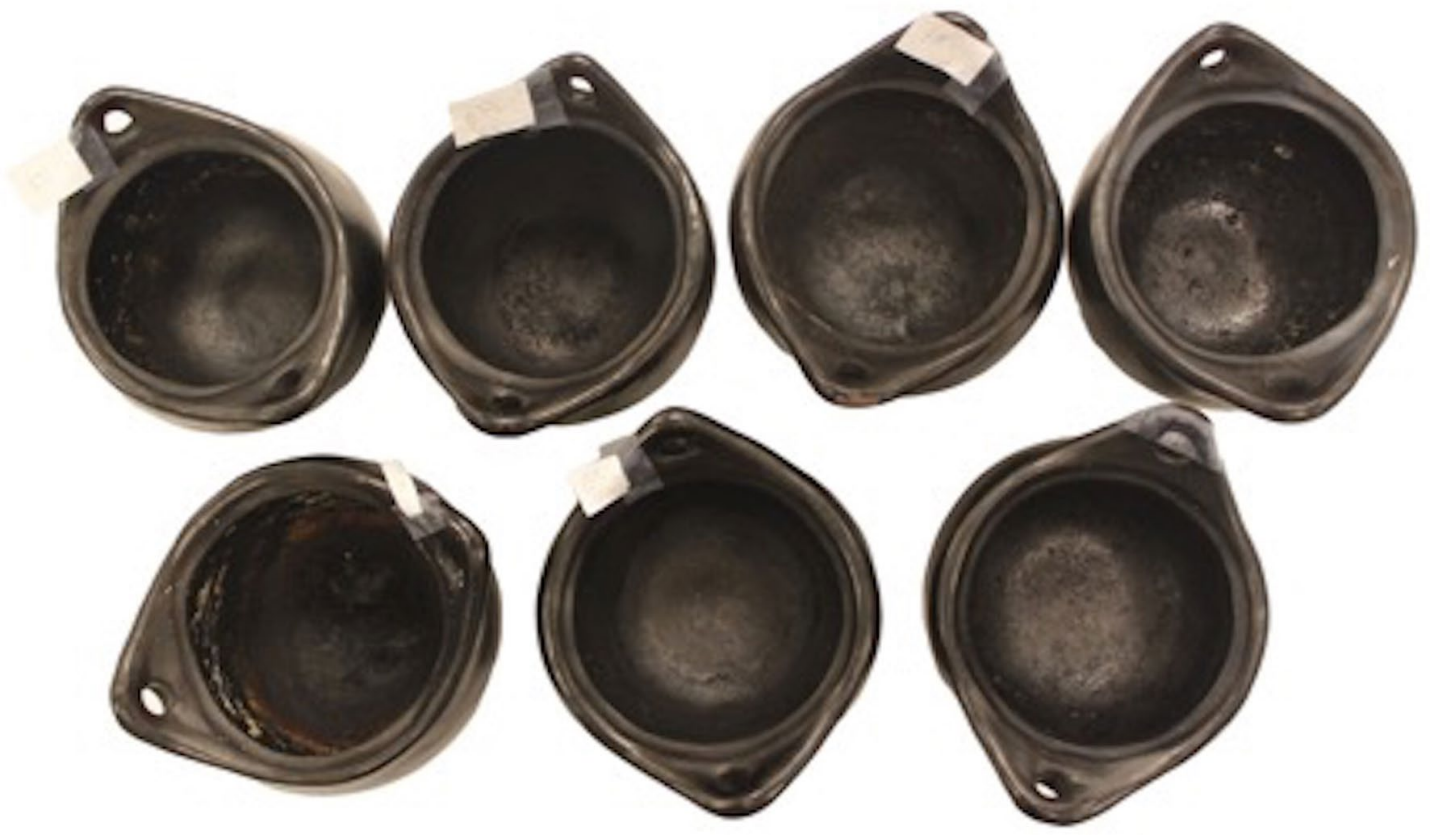
Unglazed “La Chamba” pottery used for each cooking experiment; photo taken after the final cooking event (carbonized organic patina residue and charred macro-remains are visible inside the pots).

The overall cooking experiment was performed over the course of one year, and comprised seven cooking experiments, with periodic charring episodes to mimic the conditions under which some archaeological food residues are recovered (primarily as carbonized residues; Fig. 1; Table 1). After 50 replicates of cooking the same primary recipe (part one of the experiment), the foodstuffs (recipes) cooked within the vessels were changed for 1 to 4 final cooking events to study how the surface and absorbed residues changed in their chemical compositions.

**Table 1:** Recipe information for each experimental condition.

Experiment	PRIMARY RECIPE (1): Cooked 50 times Charred macro-remains samples (n = 8) Carbonized thin-layer residue sample (n = 1)	FINAL RECIPE (2): Cooked 1–4 times Charred macro-remains samples (*n = 1 or 2) Carbonized thin-layer residue samples (**n = 1 or 2)
FB	FB_1_ whole maize kernels (1/2 cup)	FB_2_ wheat flour (1/2 cup) *n = 2 **n = 2
KV	KV_1_ maize flour (1/2 cup)	KV_2_ wheat flour (3/8 cup) + deer meat (1/8 cup) *n = 1 **n = 1
MM	MM_1_ maize flour (1/2 cup)	MM_2_ maize flour (3/8 cup) + deer meat (1/8 cup) *n = 1 **n = 1
GT	GT_1_ hominy (1/2 cup)	GT_2_ wheat flour (1/2 cup) *n = 2 **n = 2
AM	AM_1_ hominy (3/8 cup) + deer meat (1/8 cup)	AM_2_ hominy (3/8 cup) + wheat flour (1/8 cup) *n = 1 **n = 1
CH	CH_1_ wheat flour (1/4 cup) + maize flour (1/4 cup)	CH_2_ maize flour (1/2 cup) *n = 2 **n = 2
JS	JS_1_ wheat flour (3/8 cup) + maize flour (1/8 cup)	JS_2_ maize flour (1/2 cup) *n = 2 **n = 2

Two cereals were selected as the basis for these recipes due to their global archaeological significance as well as their distinct carbon isotope values: maize (*Zea mays* L.; a C_4_-plant) and wheat (*Triticum* spp. L.; a C_3_-plant). To explore the impacts of more complex recipes on carbonized and absorbed residue chemical and isotopic composition, ingredients were prepared in several combinations (Table 1). Wheat was prepared as a flour, maize was prepared in 3 different ways (whole kernels, ground maize flour, and hominy), and 3 recipes included deer meat. The bulk carbon and nitrogen stable isotope values of carbonized surface residues and the absorbed lipid compositions of the pottery fabric were determined from samples collected throughout the year-long cooking experiment. Comparison of the bulk stable carbon and nitrogen isotopic composition of the carbonized residues, and the biomarker compositions and compound-specific stable carbon isotopic values of the absorbed lipid residues, to the reference materials allowed us to trace and identify the extent of changes in the composition of the different residues.

## Results

### Bulk stable carbon and nitrogen isotope analysis of recipe ingredients

Our ingredient reference materials exhibited *δ*^13^C values within the expected ranges for C_3_ and C_4_ plants, and a wild herbivore (Table 2). Few studies have focused on the chemical differences between hominy and untreated maize, and those that have primarily focused on changes to bulk carbon values^48–51^. As previously observed by Marino and DeNiro^48^^,^ the carbon isotope values for maize and hominy (maize prepared with calcium hydroxide, Ca(OH)_2_, also known as lime) are essentially the same, indicating the lime treatment of maize does not significantly alter the *δ*^13^C value (maize/hominy *δ*^13^C = – 11‰). Lovis et al.^49^ found that hominy prepared by combining maize with hardwood ash (carbonized C_3_ wood used as the alkaline component) could potentially alter *δ*^13^C values of resulting residues if the hardwood ash was not completely removed from the mixture (but they found no change in *δ*^13^C values of the resulting hominy maize kernels alone). Previous studies have not explicitly attended to nitrogen isotope effects but our results show maize and hominy have slightly different *δ*^15^N values (maize =  + 3.8‰, hominy =  + 4.4‰), suggesting that the process of creating hominy may have altered the nitrogen isotope ratio of the original maize and caused slight ^15^N enrichment in the hominy product. Similarly, Warinner^52^ reports no difference in *δ*^13^C values between raw and nixtamalized maize but found similar changes to *δ*^15^N values: raw dried maize average *δ*^15^N value =  + 3.6‰ ± 0.8‰, while the nixtamal maize average *δ*^15^N value =  + 4.2‰ ± 0.4‰, showing the same ~ 0.6‰ offset between untreated and lime-treated maize as our experiment. Alkaline processing of maize is known to alter the protein structure of the resulting hominy (amongst other changes), perhaps shifting the isotopic composition, which in turn may have downstream effects on organic residues and consumer isotopic values; these aspects of nixtamalization should be considered and studied further^51,53^.

**Table 2:** Carbon and nitrogen bulk isotope values for raw ingredients used in this study (not cooked, not carbonized).

Ingredient	*δ*^13^C (‰)	*δ*^15^N (‰)
Maize flour	− 11.2	+ 3.8
Hominy	− 11.1	+ 4.4
Wheat flour	− 26.4	+ 1.4
Deer meat	− 26.9	+ 4.9

Note that the same maize kernels were used in whole kernel, flour, and hominy preparations.

The wheat flour has a *δ*^13^C value typical for a C_3_-plant (– 26.4‰) and has the lowest *δ*^15^N value at + 1.4‰. The difference in *δ*^15^N values between maize and wheat largely relates to the different environments where these crops were grown (e.g. soil conditions, nutrient availability, aridity, fertilizer effects, etc.)^54–56^. The deer meat *δ*^13^C value is consistent with a wild deer diet of C_3_ plants (*δ*^13^C = – 26.9‰). The deer meat displayed the highest *δ*^15^N value (*δ*^15^N =  + 4.9‰) which reflects the isotopic average of the C_3_ plants the deer consumed during life plus a trophic enrichment factor of ~  + 3–5‰^57,58^.

### Bulk isotopic analysis of charred macro-remains

#### *Variations in bulk* δ^13^C *values of charred macro-remains*

**Figure 2:**
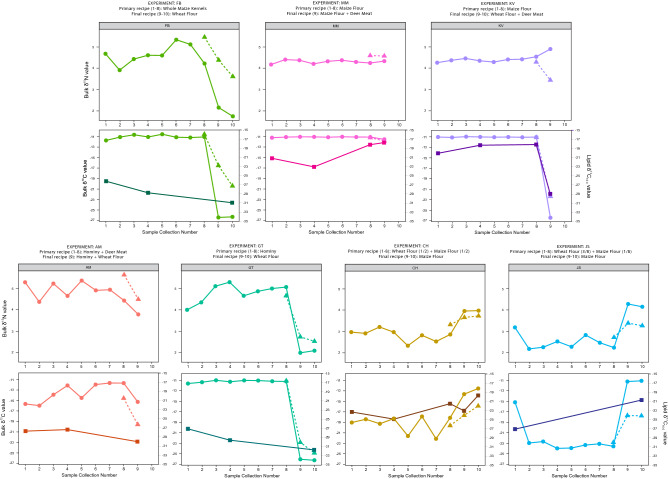
Isotope results for bulk stable carbon and nitrogen of charred macro-remains, carbonized thin-layer organic patina residues, and compound-specific carbon isotope analyses of absorbed lipids. The macro-remains are represented as circles. The first 8 macro-remains are from the primary recipe cooking events (sample collections 1–8); The recipes were changed for the final cooking events (sample collections 9–10). The organic patina residues are represented as triangles. Patina residue #1 was collected with the final primary recipe cooking event (sample collection 8) followed by patina residues which were collected after the recipe changed (sample collections 9–10). Four experiments did not have sufficient ingredients for the final replicate of the changed recipe and therefore their sample collections end with sample collection 9. Compound-specific δ^13^C values for the C_16:0_ fatty acids from lipid extracts from the cooking experiment pottery results are represented by squares.

The *δ*^13^C and *δ*^15^N values of the charred macro-remains from the burned foods cooked over the experimental timeframe are reported in Table 3 and Fig. 2. Overall, the charred macro-remains show close concordance with the *δ*^13^C values of their respective recipe ingredients^59,60^. Charred macro-remain *δ*^13^C values also remain consistent with ingredient *δ*^13^C values after the recipe was changed (Fig. 2). This is most pronounced in experiments where the final recipe cereal ingredients were changed from wheat (C_3_) to maize (C_4_) or vice versa (see FB, KV, GT). For example, *δ*^13^C values of carbonized macro-remains for experiment GT shifted from – 11 to – 26‰, concurrent with the shift from a 100% hominy to a 100% wheat-based recipe.

**Table 3:** Bulk carbon and nitrogen and compound-specific carbon isotope values for each experimental condition and carbonized sample type (macro-remains, organic patina residue, and absorbed lipid residue samples).

Experiment	Sample collection number	Sample ID	Recipe	Sample type	δ ^13^C (‰)	δ ^15^N (‰)	St dev of δ^13^C	St dev of δ^15^N	No. of replicates analyzed	Absorbed lipid δ ^13^C_16:0_ (‰)
AM	1	AM_1	Hominy (3/8) + deer (1/8)	Macro-remains	− 15.7	5.3	0.4	0.1	2	− 27.8
AM	2	AM_2	Hominy (3/8) + deer (1/8)	Macro-remains	− 16.0	4.4				
AM	3	AM_3	Hominy (3/8) + deer (1/8)	Macro-remains	− 13.9	5.2				
AM	4	AM_4	Hominy (3/8) + deer (1/8)	Macro-remains	− 12.1	4.7	0.2	0.1	2	− 27.4
AM	5	AM_5	Hominy (3/8) + deer (1/8)	Macro-remains	− 14.5	5.4	0.6	0.1	2	
AM	6	AM_6	Hominy (3/8) + deer (1/8)	Macro-remains	− 12.0	4.9	0.1	0.1	2	
AM	7	AM_7	Hominy (3/8) + deer (1/8)	Macro-remains	− 11.7	5.0	0	0.4	2	
AM	8	AM_8	Hominy (3/8) + deer (1/8)	Macro-remains	− 11.7	4.4	0.1	0.1	2	
AM	8	AM_8r	Hominy (3/8) + deer (1/8)	Thin-layer residue	− 14.6	5.7	0.1	0.1	2	
AM	9	AM_9	Hominy (3/8) + wheat flour (1/8)	Macro-remains	− 15.3	3.8	0.7	0.3	3	− 30.1
AM	9	AM_9r	Hominy (3/8) + wheat flour (1/8)	Thin-layer residue	− 19.6	4.5	0.1	0.1	3	
CH	1	CH_1	Maize flour (1/4) + wheat flour (1/4)	Macro-remains	− 19.0	3.0				− 23.4
CH	2	CH_2	Maize flour (1/4) + wheat flour (1/4)	Macro-remains	− 18.4	2.9				
CH	3	CH_3	Maize flour (1/4) + wheat flour (1/4)	Macro-remains	− 19.3	3.2				
CH	4	CH_4	Maize flour (1/4) + wheat flour (1/4)	Macro-remains	− 18.3	3.0				− 24.9
CH	5	CH_5	Maize flour (1/4) + wheat flour (1/4)	Macro-remains	− 21.6	2.3	0.1	0	2	
CH	6	CH_6	Maize flour (1/4) + wheat flour (1/4)	Macro-remains	− 17.9	2.8	0.1	0.1	2	
CH	7	CH_7	Maize flour (1/4) + wheat flour (1/4)	Macro-remains	− 22.1	2.5	0.2	0.4	2	
CH	8	CH_8	Maize flour (1/4) + wheat flour (1/4)	Macro-remains	− 18.1	2.9				− 21.6
CH	8	CH_8r	Maize flour (1/4) + wheat flour (1/4)	Thin-layer residue	− 19.6	3.3	0.1	0.1	3	
CH	9	CH_9	Maize flour	Macro-remains	− 13.6	4.0				− 23.1
CH	9	CH_9r	Maize flour	Thin-layer residue	− 17.7	3.7	0.1	0.3	3	
CH	10	CH_10	Maize flour	Macro-remains	− 12.5	4.0				− 19.8
CH	10	CH_10r	Maize flour	Thin-layer residue	− 15.9	3.7	0.1	0.1	2	
FB	1	FB_1	Whole maize	Macro-remains	− 11.7	4.7				− 26.2
FB	2	FB_2	Whole maize	Macro-remains	− 11.1	3.9				
FB	3	FB_3	Whole maize	Macro-remains	− 10.6	4.4				
FB	4	FB_4	Whole maize	Macro-remains	− 11.1	4.6				− 28.8
FB	5	FB_5	Whole maize	Macro-remains	− 10.5	4.6				
FB	6	FB_6	Whole maize	Macro-remains	− 11.1	5.3				
FB	7	FB_7	Whole maize	Macro-remains	− 11.2	5.1				
FB	8	FB_8	Whole maize	Macro-remains	− 11.1	4.2				
FB	8	FB_8r	Whole maize	Thin-layer residue	− 10.5	5.5	0.1	0.1	2	
FB	9	FB_9	Wheat flour	Macro-remains	− 26.4	2.2	0	0	2	
FB	9	FB_9r	Wheat flour	Thin-layer residue	− 16.5	4.4	0.1	0.1	2	
FB	10	FB_10	Wheat flour	Macro-remains	− 26.3	1.7				− 30.9
FB	10	FB_10r	Wheat flour	Thin-layer residue	− 20.4	3.6	0.6	0.1	3	
GT	1	GT_1	Hominy	Macro-remains	− 11.6	4.0	0.1	0.1	2	− 27.1
GT	2	GT_2	Hominy	Macro-remains	− 11.4	4.4				
GT	3	GT_3	Hominy	Macro-remains	− 11.0	5.1				
GT	4	GT_4	Hominy	Macro-remains	− 11.3	5.3				− 29.6
GT	5	GT_5	Hominy	Macro-remains	− 11.0	4.7				
GT	6	GT_6	Hominy	Macro-remains	− 11.1	4.9				
GT	7	GT_7	Hominy	Macro-remains	− 11.2	5.0				
GT	8	GT_8	Hominy	Macro-remains	− 11.2	5.1				
GT	8	GT_8r	Hominy	Thin-layer residue	− 11.1	4.7	0.1	0	2	
GT	9	GT_9	Wheat flour	Macro-remains	− 26.1	2.0				
GT	9	GT_9r	Wheat flour	Thin-layer residue	− 22.9	2.7	0	0.3	2	
GT	10	GT_10	Wheat flour	Macro-remains	− 26.3	2.1				− 31.7
GT	10	GT_10r	Wheat flour	Thin-layer residue	− 24.9	2.5	0.1	0	2	
JS	1	JS_1	Wheat flour (3/8) + maize flour (1/8)	Macro-remains	− 15.2	3.2	0.1	0.1	2	− 27.1
JS	2	JS_2	Wheat flour (3/8) + maize flour (1/8)	Macro-remains	− 22.9	2.2				
JS	3	JS_3	Wheat flour (3/8) + maize flour (1/8)	Macro-remains	− 22.6	2.3				
JS	4	JS_4	Wheat flour (3/8) + maize flour (1/8)	Macro-remains	− 23.9	2.5	0.1	0	2	
JS	5	JS_5	Wheat flour (3/8) + maize flour (1/8)	Macro-remains	− 23.9	2.3				
JS	6	JS_6	Wheat flour (3/8) + maize flour (1/8)	Macro-remains	− 23.3	2.8				
JS	7	JS_7	Wheat flour (3/8) + maize flour (1/8)	Macro-remains	− 23.1	2.5				
JS	8	JS_8	Wheat flour (3/8) + maize flour (1/8)	Macro-remains	− 23.5	2.2	0	0.1	2	
JS	8	JS_8r	Wheat flour (3/8) + maize flour (1/8)	Thin-layer residue	− 22.8	2.7	0	2	2	
JS	9	JS_9	Maize flour	Macro-remains	− 11.2	4.3				
JS	9	JS_9r	Maize flour	Thin-layer residue	− 17.7	3.4	0	0.2	3	
JS	10	JS_10	Maize flour	Macro-remains	− 11.1	4.2				− 20.7
JS	10	JS_10r	Maize flour	Thin-layer residue	− 17.7	3.3	0.1	0.2	3	
KV	1	KV_1	Maize flour	Macro-remains	− 11.0	4.3				− 20.0
KV	2	KV_2	Maize flour	Macro-remains	− 11.1	4.4	0.1	0	2	
KV	3	KV_3	Maize flour	Macro-remains	− 10.9	4.5				
KV	4	KV_4	Maize flour	Macro-remains	− 11.0	4.4	0	0.2	2	− 18.2
KV	5	KV_5	Maize flour	Macro-remains	− 11.0	4.3				
KV	6	KV_6	Maize flour	Macro-remains	− 11.0	4.4				
KV	7	KV_7	Maize flour	Macro-remains	− 11.1	4.4				
KV	8	KV_8	Maize flour	Macro-remains	− 11.0	4.5				− 18.1
KV	8	KV_8r	Maize flour	Thin-layer residue	− 11.1	4.3	0.1	0.1	2	
KV	9	KV_9	Wheat flour (3/8) + deer (1/8)	Macro-remains	− 26.4	4.9				− 28.9
KV	9	KV_9r	Wheat flour (3/8) + deer (1/8)	Thin-layer residue	− 22.3	3.4	0.1	0.1	2	
MM	1	MM_1	Maize flour	Macro-remains	− 11.1	4.2				− 21.2
MM	2	MM_2	Maize flour	Macro-remains	− 11.0	4.4				
MM	3	MM_3	Maize flour	Macro-remains	− 11.0	4.4				
MM	4	MM_4	Maize flour	Macro-remains	− 11.0	4.2	0	0	2	− 23.1
MM	5	MM_5	Maize flour	Macro-remains	− 11.1	4.3				
MM	6	MM_6	Maize flour	Macro-remains	− 11.0	4.4				
MM	7	MM_7	Maize flour	Macro-remains	− 11.0	4.3				
MM	8	MM_8	Maize flour	Macro-remains	− 11.0	4.3	0.1	0.1	2	− 18.2
MM	8	MM_8r	Maize flour	Thin-layer residue	− 11.1	4.6	0	0.2	3	
MM	9	MM_9	Maize flour (3/8) + deer (1/8)	Macro-remains	− 11.5	4.3	0.1	0.3	3	− 17.7
MM	9	MM_9r	Maize flour (3/8) + deer (1/8)	Thin-layer residue	− 11.8	4.6	0.1	0.2	2

Charred macro-remains of recipes involving mixtures of C_3_ and C_4_ foodstuffs (e.g. CH_1_) display an isotopic signature (ca. – 18‰) consistent with a 50:50 mixture of the C_3_ (– 26‰) and C_4_ plants (– 11‰). Similarly, experiment JS_1_ comprised a recipe of 25% maize and 75% wheat, and the resulting carbonized macro-remains exhibited a *δ*^13^C value around –23‰, which is roughly equivalent to a 25% shift in *δ*^13^C value towards C_4_ plant values. This is also observed in the 75% maize, 25% deer meat mixture (AM_1_) where the carbonized residues exhibit *δ*^13^C values ranging from – 16 to – 11‰ (average *δ*^13^C = – 14‰) consistent with a 25% shift in *δ*^13^C values towards those displayed by C_3_ plants and wild deer. We observed no difference in the *δ*^13^C values of residues produced by processing maize in different forms, i.e. ground, whole and hominy.

#### *Variations in bulk δ*^*15*^*N values of charred macro-remains*

The *δ*^15^N values of the charred macro-remains generally display similar *δ*^15^N values to their respective recipe ingredients but show a slight positive offset, implying fractionation caused by heating, wherein the charred product becomes enriched in the heavier ^15^N isotope^60^. For example, experiments MM_1_ and KV_1_ used 100% maize flour, and their carbonized residues displayed average *δ*^*15*^*N* values =  + 4.3‰, which is + 0.5‰ higher than the raw maize flour value. In almost all experimental conditions we see significant changes in the charred macro-remain *δ*^15^N values after the recipe changed (sample collections 9 and 10), which are consistent with *δ*^15^N values of newly introduced ingredient(s). No change in *δ*^15^N values are observed in experiments KV_2_ and MM_2_ due to the similarity of deer and maize ingredient *δ*^15^N values (+ 4 to + 5‰). As observed for the *δ*^13^C values (above), the most pronounced changes in *δ*^15^N values occur in experiments with 100% recipe swaps (FB, GT; Fig. 2). For example, in experiment GT, charred macro-remain *δ*^15^N values shift from ~  + 4.5‰ (consistent with the cooking of 100% hominy, GT_1_) to *δ*^15^N =  + 2‰ (100% wheat-based recipe, GT_2_). Also consistent with trends in *δ*^13^C values are *δ*^15^N values of charred macro-remains from recipes with mixtures of C_3_ and C_4_ foods (CH, JS), which reflect a mixing of the recipe ingredients. For example, the mean *δ*^15^N value of CH_1_ is + 2.9‰, consistent with a 50:50 mixture of maize (*δ*^15^N =  + 3.8‰) and wheat (*δ*^15^N = 1.4‰) plus a slight increase in ^15^N likely due to heating effects. After the recipe swap, the *δ*^15^N values of charred macro-remains from CH_2_ became more elevated (*δ*^15^N =  + 4‰), which is consistent with the recipe switch to 100% maize. For experiment AM, we only observed a slight change in *δ*^15^N values when deer (AM_1_) was replaced with wheat (AM_2_), despite their difference in *δ*^15^N values; there was only a slight depletion from + 4.9 to + 3.8‰ after the change from 75% hominy/25% deer to 75% hominy/25% wheat flour. This is likely because hominy has a high *δ*^15^N value similar to the deer, and in AM_2_ hominy is still the dominant ingredient and therefore skews the *δ*^15^N value of the charred macro-remains sample towards that ingredient.

### Carbonized thin-layer organic patina residue isotopic results

The thin, organic patina carbonized residues from the interior of each pot (the residue layer in most intimate (direct) contact with the pot’s inner surface, often observable on archaeological pottery) show a more complex picture of cooking events and the relationship between original recipe ingredients (Fig. 2; Table 3). The stable carbon and nitrogen isotopic values indicate that these residues reflect a combination of the final ingredients cooked in the vessel and traces of ingredients from previously cooked recipes. Unlike the charred macroscopic food residues, which retained the isotopic values of their most recent ingredients and shifted with each recipe change, the thin-layer organic patina residues more closely resembled a carbonized recipe palimpsest: the *δ*^13^C and *δ*^15^N values predominantly reflect the final recipe but chemically hints of the previous recipe remain. Our initial hypothesis was that patina residue 1 (corresponding to charred macro-remain sample collection 8) would accurately reflect the original recipe ingredients and that subsequent sampling of this patina residue layer after the recipe change (sample collections 9 and 10) would display a complete chemical shift (“over-write”) to the isotopic values of the new ingredients. Instead, we see more variability in this carbonized patina residue, with values reflecting both the final ingredients and previous ingredients prepared in the pot (Fig. 2). Therefore the patina residues appear to retain more of the chemical evidence of previous cooking events than the charred macro-remains (despite the macro-remains cooking directly on top of this thin, directly adhering layer). For the few experiments where 3 organic patina carbonized residues were collected (FB, GT, CH, JS) we can see a time-lapse in effect on *δ*^13^C and *δ*^15^N values changing across those three residues: patina residue 1 (sample collection 8) closely reflects the primary recipe isotopic values, patina residue 2 (sample collection 9) has intermediate isotope values but is generally closer to the final recipe, and patina residue 3 (sample collection 10) is closer still to the final recipe ingredients. For example, experiment GT_1_ began with 100% hominy, and patina residue 1 (sample collection 8r) displays *δ*^13^C and *δ*^15^N values that are very close to those of the charred macro-remains (and therefore the original raw ingredient isotope values). Between collection 8 and 9, experiment GT_2_ switches recipes to 100% wheat flour (an ingredient shift from C_4_ to C_3_) and patina residue 2 (collected after the first GT_2_ cook, sample collection 9r) displays significantly lower *δ*^13^C and *δ*^15^N values, though not as low as the charred macro-remains (Fig. 2). Thus, the carbonized thin-layer patina residues retained some of the material composition from the previous C_4_ hominy cooking events alongside new inputs from C_3_ wheat flour. GT patina residue 3 (sample collection 10r) displays further reduction in *δ*^13^C and *δ*^15^N values, this time with the effect more significant in *δ*^13^C value (Fig. 2). At the conclusion of the experiment, stable isotope values from the final organic patina residue still did not match those of the charred macro-remains of the final ingredients, suggesting there is a longer chemical “lag-time” (memory) in formation of this thin-layer residue compared to the most recent cooking activities.

### Isotopic mixing model (IsoSource) results

We ran the isotopic results from the charred macro-remains and the organic patina residues through a basic mixing model (IsoSource v1.3.1^61^) to see how the bulk isotopic values would be interpreted given the known ingredients (sources) and known quantities in the recipes (see Supplementary Methods and Supplementary Table S1 online). We found that for most of the experimental conditions, the mixing model generally estimated the ingredients in proportions close to the original recipes. For example, using IsoSource to model the macro-remains for GT_2_ and FB_2_ experiments (recipes comprised 100% wheat after months of cooking 100% hominy/maize), we see that those residues were estimated to be 95–100% wheat and only 0–5% hominy/maize (given both sources as potential contributors, see Supplementary Methods online). The IsoSource mixing models also generally returned good estimations for recipes with C_3_/C_4_ mixtures (CH, JS, AM). For example, the JS_1_ recipe comprised 3:1 wheat to maize, and the mixing model generally estimated 75–80% wheat and 20–25% maize for those corresponding macro-remains. The JS_2_ recipe then switched to 100% maize, and the resulting mixing model estimates for those macro-remain samples were 95–100% maize and 0–5% wheat.

For the organic patinas sampled after the recipes were changed, the bulk isotope values showed that these residues reflected a mixture of ingredients appearing as a palimpsest, and the mixing model results also support this, showing proportional changes that indicate retention of earlier ingredients despite those ingredients not being part of the final cooking events (See Supplementary Table S1 online). For example, the FB_1_ recipe comprised 100% maize kernels, and the first organic patina sample (FB_8r) was estimated by IsoSource at 95–100% maize and 0–5% wheat, (given those two potential sources). The recipe then changed for FB_2_ to 100% wheat, and the following patina residue (FB_9r) was estimated to have 65–75% maize and 25–35% wheat, followed by further cooking of the FB_2_ recipe and another organic patina sample (FB_10r), which was estimated to have 30–45% maize and 55–70% wheat. We see the declining influence of the previous ingredient (maize from FB_1_) in the organic patina residue over time. Therefore, the mixing model results also support the interpretation that patinas reflect multiple cooking events and do not reflect the very final ingredients prepared in the pot. We did find that deer meat was over-estimated by the mixing model when it was a source along with wheat (see AM and KV in Supplementary Table S1 online), which was likely exacerbated by the fact that the deer and wheat sources possessed very similar carbon isotope values. Mixing models work best when sources are well-defined and isotopically distinct. Further experiments creating residues from combining resources that have similar isotopic values would be useful to test the limits of mixing model quantification and source discrimination.

### Organic residue analysis of absorbed lipids

Acid-extracted lipids from the pottery fabric were analysed by gas chromatography (GC) and GC-mass spectrometry (GC–MS) to enable quantification and identification. Lipids were recovered from 24 of the 32 cooking experiment total lipid extracts (TLEs) analysed (75%). Low lipid recovery was likely a result of two factors; the lipid content of plant-based food generally is at least tenfold lower than that of meat, and plant lipids are poorly mobilised resulting in limited transfer to the pot matrix (see Hammann and Cramp^33^). Heavy burnishing on the interior surface of the vessel may also have intensified this by interfering with the absorption of lipids into the pottery fabric, further experimental work should examine different pottery treatments to better characterize these relationships^62^. The TLEs from wheat-based experiments were dominated by C_16:0_, C_18:1_ and C_18:2_ fatty acids (see Supplementary Figs. S1 and S2 online). In maize-based experiments the TLEs were dominated by the unsaturated C_18:2_ and C_18:1_ fatty acids (Supplementary Figs. S1 and S2 online). Plant-derived biomarkers including sterols, *n-*alkanes of carbon chain length C_21_ to C_25_ and long-chain fatty acids (LCFA) of carbon chain length C_14_ to C_30_ were detected in the majority of the TLEs. Due to low concentrations of C_16:0_ and C_18:0_ fatty acids it was not possible to determine stable carbon isotope values for all lipid components. Therefore, C_16:0_ fatty acid was chosen to determine the isotopic composition of the absorbed residues as it is common to all food ingredients and would enable the contributions of C_3_ and C_4_ plant lipids to the TLEs to be clearly distinguished.

#### Compound-specific carbon isotopic analyses

The TLEs were subsequently analysed using GC-combustion isotope ratio-MS (GC-C-IRMS) in order to determine the isotopic composition of the absorbed lipids (Table 3). In order to provide a baseline from which interpretations could be made, reference materials were analysed in conjunction with the pottery sherds (Supplementary Table S2 online). The ground maize and maize kernels displayed *δ*^13^C_16:0_ values of – 17.7‰ and – 19.4‰, respectively. Hominy was less enriched in ^13^C than both the ground and whole maize (*δ*^13^C_16:0_ value of – 23.6‰). This was likely due to changes taking place during the nixtamalization process but it is unclear how this has occurred^48,49^.

The wheat kernel and ground wheat displayed *δ*^13^C_16:0_ values of –34.2 ‰ and –33.4 ‰, respectively. The deer meat *δ*^13^C_16:0_ value was –33.5 ‰ which is consistent with the animal having lived in a C_3_ environment. The *δ*^13^C values of the C_16:0_ fatty acids from cooking experiments with an 100% C_4_ to C_3_ recipe swap (FB and GT) exhibit a shift in *δ*^13^C values towards the values of the wheat cooked after the recipe swap (Fig. 2). Interestingly, the *δ*^13^C_16:0_ values of the hominy and maize cooked in experiments GT and FB, respectively, do not correspond to the isotopic values of the reference materials but instead exhibit lower *δ*^13^C values (Fig. 2). Here, these differences could be a reflection of only the surface wax lipids mobilizing in the water during cooking of the kernel compared to the maize flour, which would contain both the surface wax and endosperm lipids. The acidified methanol extraction of the maize kernel itself would penetrate the epicuticular layer results in a *δ*^13^C value corresponding to that of maize flour.

This isotopic difference is also observed in experiments with mixtures of foods of differing isotope values (e.g. CH) where the 50% wheat, 50% maize mixture does not have a consistent isotopic value across time (Table 3). This is likely due to the low extraction efficiency of water for cereal lipids resulting in low concentrations of lipid being incorporated into the ceramic fabric ^33^. The most pronounced shift in isotopic composition was observed in experiment KV, where a 100% maize-based recipe was swapped to a 75% wheat, 25% deer mixture. Here, a shift from – 18 to – 29‰ was observed. This suggests that the lipids from the cooking of maize are being retained in the vessel fabric as the *δ*^13^C value of the palmitic acid in the wheat and deer mixture has not quite reached that of the reference material (– 33‰).

The results from the compound-specific analyses of the absorbed lipid residues demonstrate that lipids retained within the ceramic fabric reflect the lipid composition of commodities cooked within the vessel. A shift in the isotopic composition of *δ*^13^C_16:0_ values across the first three fabric samples in experiments (e.g. KV, MM, CH) indicates that the lipids are incorporated into the vessel wall over time as the *δ*^13^C values do not immediately reflect their reference materials (ingredients) after the initial cook, but instead shift towards the *δ*^13^C_16:0_ of the ingredients from fabric sample 2 onwards. After the recipe swap the *δ*^13^C_16:0_ values show a small but gradual shift towards the isotopic value of the “new” foodstuffs. The more extreme shift in *δ*^13^C_16:0_ value observed in experiments containing deer meat is likely due to the higher lipid content of meat relative to plants, making this overprinting more pronounced^30,63^.

### Characteristic lipid patterns in maize and wheat

**Figure 3:**
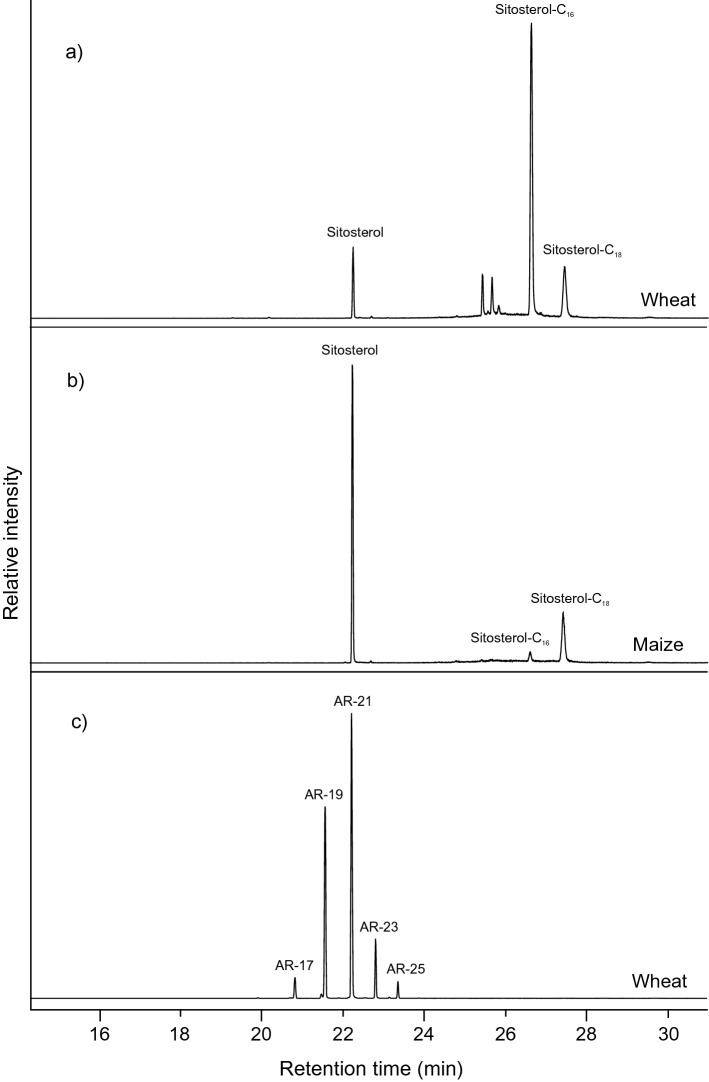
Characteristic cereal biomarker lipids from the reference materials. Where (**a**) shows the trimethylsilyated sitosterol and sitosterol fatty acid esters in the wheat displayed using the extracted ion trace of *m/z* 396.3756; (**b**) shows the trimethylsilyated sitosterol and sitosterol fatty acid esters from the maize displayed using the extracted ion trace of *m/z* 396.3756 and (**c**) shows the alkyl resorcinols present in the reference wheat displayed using the extracted ion trace of *m/z* 268.1315.

Total lipid extracts of maize and wheat were analysed by GC and GC/Quadrupole-Time of Flight MS (GC/Q-ToF MS) in order to identify characteristic lipids that could be used for their identification in the absorbed residues from the cooking experiments (Table 4). Recently, plant sterols and alkylresorcinols have been explored as potential biomarker compounds for the identification of cereal (including wheat) processing in archaeological pottery^33^. The characteristic cereal lipids were identified in reference wheat (Fig. 3a,c) by displaying the extracted ion traces of *m/z* 268.1315 (alkylresorcinols) and 396.3756 (trimethylsilylated sitosterol and sitosterol fatty acid esters). Maize did not contain any alkylresorcinols, yet, contained plant sterols identical to those detected in the reference wheat, making analytical discrimination challenging. However, it was found that both the ratio of free to esterified sitosterol and the ratio of C_16_ to C_18_ fatty acid esters was distinctly different in wheat and maize (Fig. 3a,b). Wheat featured a dominance of sitosterol fatty acid sterol esters over free sitosterol and about a fourfold higher abundance of C_16_ fatty acid sterol esters compared to C_18_ fatty acid sterol esters. Contrastingly, free sitosterol was more abundant than the fatty acid esters in maize and the C_18_ fatty acid sterol esters dominated the sterol ester pattern (ca. eightfold more abundant than C_16_ fatty acid esters). Both the presence (or absence) of alkylresorcinols and the pattern of free and esterified sitosterol were used to identify absorbed lipids deriving from wheat or maize in ceramics from cooking experiments.

**Table 4:** Distribution of characteristic lipid biomarkers in selected TLEs and reference material analysed by GC/Q-ToF MS.

Experiment	Description	Alkylresorcinols	Sitosterol free:esterified	Sitosterol ester C_16_:C_18_
GT #8	100% hominy	n.d	5:1	20:100
GT #10	100% wheat	Present	1:1	100:20
JS #8	75% wheat, 25% maize	n.d	2:1	100:20
JS #10	100% maize	n.d	3:1	100:80
FB #8	100% maize	n.d	Only free	No esters detected
FB #10	100% wheat	Present	1:1	100:15
Reference wheat	100% wheat	Present	1:4	100:25
Reference maize	100% maize	n.d	5:1	15:100

*n.d.* not detected.

#### Analysis of lipid patterns in absorbed lipid residues in ceramic fabric from cooking experiments

The analysis of TLEs from the cooking experiments revealed that traces of the target lipid compounds were absorbed during the cooking processes. More importantly, the lipid patterns corresponded very well to the patterns expected from the analysis of the reference plants. For example, in TLE FB #8 (100% maize, sample collection 8) no alkylresorcinols and only free sitosterol were detected, which reflected the absence of alkylresorcinols and low proportions of esterified sitosterol in the reference maize (Table 2; Supplementary Fig. S3 online). After the recipe had changed to 100% wheat (FB #10, sample collection 10), sitosterol esters were clearly detectable at similar intensity as free sitosterol (Supplementary Fig. S3 online) and the ratio of C_16_ to C_18_ esters of ca. 100:15 was comparable to the ratio observed in the wheat reference. In the TLE JS #8 (75% wheat, 25% maize, sample collection 8) the ratio of free to esterified sitosterol was about 2:1, which clearly represented a mixture of the ratios found in the pure wheat and maize references (Supplementary Fig. S4 online). When the recipe was changed to 100% maize (JS #10, sample collection 10) the ratio increased to 3:1; approaching the ratio of pure maize (5:1; Supplementary Fig. S4 online). Similar observations were made in the ratio of C_16_ to C_18_ sterol esters, where a ratio of 100:20 initially observed, shifted to a higher proportion of C_18_ esters (100:80) after the recipe change. Finally, the biomarker pattern observed in the TLE from the cooking experiment using 100% hominy (GT #8, sample collection 8) corresponded with the pattern observed in the maize reference runs (Supplementary Fig. S5 online); after the recipe change to 100% wheat (GT #10, sample collection 10) the biomarker ratios shifted towards wheat-typical values including the presence of detectable quantities of alkylresorcinols (Supplementary Fig. S5 online).

## Discussion

Comparing the respective changes in isotopic composition of the charred macro-remains, carbonized thin-layer patina residues, absorbed lipid residues, and the presence/absence of characteristic plant lipids across the year-long cooking experiment has enabled a better understanding of absorbed and surface residues during the use-life of ceramic cooking vessels^46,47^.

The *δ*^13^C and *δ*^15^N values of charred macro-remains generally reflect the ingredients of the final foodstuffs that were cooked in the vessel. After changing recipes, *δ*^13^C values of these burned food crusts accurately reflect the ingredients of the new recipe. The *δ*^15^N values also shift towards but do not entirely reach the values of the new recipe. Bulk analyses of charred macro-remains therefore closely represent the last foods that were cooked within the vessel rather than an accumulation of the foodstuffs cooked over the lifetime, or recent life, of the pot. These findings are in accordance with other studies of bulk *δ*^13^C and *δ*^15^N values from charred plants^59,64^. Early work by DeNiro and Hastorf^59^ indicated that charred ancient plant remains retained their isotopic values (within ~ 3‰ compared to modern analogous plants), and further studies by Fraser et al.^60^ and Styring et al.^65^ and others, found no significant systematic changes to *δ*^13^C values in charred cereals and pulses, and small (< 1‰) changes to *δ*^15^N values. Recently, Szpak and Chiou^66^ found that under particularly favourable taphonomic conditions, some desiccated but uncharred ancient plants may also retain their biogenic bulk isotopic values. Further studies of organic residues recovered from environments such as those of the north coast of Peru may indicate that those conditions are favourable for the survival not only of bulk isotopic values but of many resource biomarkers despite hundreds to thousands of years of time elapsing^66^.

Unlike the charred macro-remains, the *δ*^13^C and *δ*^15^N values of carbonized adhering organic patina residues are far less likely to represent only the final ingredients cooked in the pot. We believe this would be particularly true in culinary contexts where individual vessels have multiple purposes, or where a wide range of ingredients (C_3_, C_4_, terrestrial, aquatic) are utilized in a variety of combinations over the use-life of the vessel. While the patina residues are influenced by the final foods cooked, these patina residues may also retain chemical signatures of ingredients from previous cooking/charring events. It is therefore critical for archaeologists to carefully consider the use life of a ceramic vessel and its surrounding ecological context: not all superficial carbonized residue layers—and indeed likely very few—reflect a single depositional charring event (i.e. the last supper).

Lipid biomarker analysis demonstrates that the lipids absorbed into the vessel wall correspond to a gradual overprinting of the lipids from previous cooking events. Notably, although the ratios of different lipid components clearly shifted after a recipe change, they did not fully reach the typical values of the pure newly added foodstuff, even after several additional cooking events. This demonstrates that a slow replacement of lipids took place over sequential cooking events, but the absorbed lipids still represented a mixture of the commodities cooked over the history of vessel’s use and were not only indicative of the most recently processed foodstuffs. Further experiments are required to determine whether lipids are replaced entirely with subsequent cooking events.

In summary: (1) bulk stable isotope analysis of carbonized food macro-remains represent the last foods cooked within the vessel; (2) bulk stable isotope analysis of the carbonized adhering organic patina residues represent a palimpsest of previously cooked ingredients but are strongly skewed towards the final recipe. The stable isotope values of these organic patina residues were slower to change over time than the more substantial carbonized macro-remains; (3) the absorbed lipids within the ceramic matrix are not immediately replaced by the new recipes. Instead, lipids are replaced slowly, over time, and represent a mixture of the ingredients cooked during the vessel’s use-life. These three different residue types reveal cooking events across different time scales: the lipids absorbed into the pottery walls reveal the pot’s use history, the carbonized surface thin-layer patina shows a mixture of the most recent few cooking events, and the most superficial samples, the charred macro-remains, show the final snapshot of the ingredients last cooked (burned) in that vessel.

We propose that these different residue forms present unique opportunities for archaeologists to study the various resources that may have been used across multiple time-scales within a cooking vessel’s use history. The chemical data from these residues highlight different dietary components (emphasizing carbohydrates and proteins in surface residues and lipids in absorbed residues), and therefore using them in conjunction offers a potentially more robust and more complex picture of ancient food practices. Most studies of organic residues have used biomarkers in the qualitative sense (i.e. using presence/absence of specific compounds to indicate the inclusion of particular ingredients in the past) without significant reflection or interrogation of the timescale and events under which those residues were formed, and whether or not they represent the lifetime use of the pot^20,44^. However, experiments such as this one contribute to our ability to quantitatively understand residues better: known ingredients prepared in specific proportions help us understand the resulting residue data. This knowledge can then be applied in archaeological settings (acknowledging caveats such as equifinality and biomarker specificity, etc.). Our results indicate that charred macro-remains have the potential to tell us both qualitative and quantitative recipe ingredient information, while absorbed residues demonstrated more timescale variability and are thus more amenable to qualitative interpretations. This information could be particularly important in estimating the lifespan of a pottery vessel (of which very little is known) especially if used in conjunction with compound-specific radiocarbon dating of both absorbed lipid and carbonized residues^67^.

The residues tested here were derived from a subset of potential cooking, processing, and storage practices that people utilize, and this research specifically focused on residues formed within one material type (ceramics) by specific methods, boiling and “accidental” charring, and for a predetermined use life (1 year). Therefore, the results presented here may be specific to residues created in clay pots over a particular duration, and residues extracted from other materials or formed under other preparation methods may or may not follow the same patterns. For example, we still do not know how many cooking events are required to completely change the lipid signature of a pot and thus cannot differentiate between a pot that has been used to process one foodstuff its entire life or if its function was changed and the lipids completely replaced. Further experimental studies are recommended to understand how residues form on other materials and under different conditions (such as fermentation, roasting, long-term storage, etc.) over variable periods of time.

The findings we report here indicate the value of experimental archaeological projects, and additional studies should expand on the range of ingredient combinations (such as inclusion of diverse C_3_ foods within the same recipe and mixtures of terrestrial and aquatic foods) to provide further qualitative and quantitative information for interpreting organic residues from archaeological specimens. We hope that future studies examining archaeological pottery sherds and other materials will aim to retain these various residue deposits (which often means leaving pottery and other materials unwashed, to best preserve adhering and absorbed surface residues) so as to further unlock these different scalar residue records and potentially provide greater insights into human subsistence practices and resource use in the past.

Combined isotopic and biomarker analyses on both carbonized and absorbed residues from the same vessel can refine archaeological interpretations regarding vessel use and culinary practices by providing a chemical signature of the ingredients used in the pot’s final meal (*δ*^13^C and *δ*^15^N values from carbonized macro-remains) as well as a mixture of previously utilized ingredients (carbonized organic patina residue *δ*^13^C and *δ*^15^N values and absorbed lipid residues). These results in turn can complement other lines of archaeological and paleoenvironmental evidence including faunal, macro-and micro-botanical remains to provide new insight not only into ancient culinary practices, but also how past food procurement and production strategies integrated and adapted to local environments. Past human subsistence strategies have generated lasting, interconnected transformations to global environments^68–72^, and archaeological studies of human foodways through residue analyses can make important contributions to understanding resource use in the past. Interpreting archaeological dietary proxies requires careful consideration of material use-life including pre- and post-depositional processes. Controlled “middle-range” studies, particularly long-term experiments designed to mimic the full duration of an object’s use-life, enhance our interpretations of the ‘residues’ of human activities left behind in the archaeological record.

## Methods

The grains utilized in this experiment were USDA Certified Organic dried whole maize kernels (*Zea mays* L.) and USDA Certified Organic whole hard red winter wheat kernels (*Triticum aestivum* L.). Maize and wheat grains were ground to flour in a (new, washed) grain mill (NutriMill). Portions of the maize kernels were left whole and others were prepared as hominy. The hominy processing involved soaking whole maize kernels with water and food-grade lime (calcium hydroxide, Mrs. Wages pickling lime) for 8 h, warming the mixture over low heat for 4 h, rinsing the hominy in water, followed by transferring into plastic bags which were frozen and used over the course of the experiment. Meat from a young deer (*Odocoileus hemionus californiensis*) was donated by the University of California, Santa Cruz (UCSC) Laboratory of Anthropology, from which 7 cups (1.5 kg) of venison were utilized (taken from different parts of the body of the deer). All dry ingredients were stored under cool and dry conditions, while the venison and hominy were stored in a freezer until required for cooking. Uncooked primary ingredients were reserved for chemical analyses and included ground maize, ground wheat, hominy and deer meat.

All recipe information is provided in Table 1. Each of the 7 recipes were cooked (between 55 and 85 °C) with ½ cup water (125 mL) in “La Chamba” pottery vessels (unglazed but the pots were burnished on their interior and exterior surfaces) for 1 h periods once a week over the course of 50 weeks before the recipe was changed for a final, short cooking period (Fig. 1). Between cooking events the foodstuffs were gently cleaned out of the pots using water and an apple wood stick to wipe clean the interior (no vigorous surface scrubbing was undertaken), and no detergent was used during the cleaning process, only water. To mimic the carbonized residues recovered from archaeological contexts, every 7^th^ week during cooking the foodstuffs were intentionally burned until a blackened layer was formed on the interior of the vessel. Charred food macro-remains from the internal base of the pot were collected in centrifuge tubes and frozen until freeze-dried for further analysis. Eight such of the carbonized food remains were collected over the 50-week period. At the 51st cooking event recipes were changed and cooked in each pot until charred. Four experiments (FG, GT, CH, JS) had sufficient ingredients to continue with the cooking regimen and in these cases the foodstuffs were cooked twice more without burning (cooking events 52, 53) followed by a cooking event in which the foods were charred and a 10th charred food residue was collected. Therefore, 8 charred food macro-remains were collected from the primary recipe and followed by 1 or 2 additional charred food crusts from the final (changed) recipe, depending on ingredient availability.

During the later stages of the experiment, it was noted that a thin, finely textured carbonized layer (organic patina) visibly adhering to the pot’s interior surface had accumulated across cooking episodes, particularly at the base of the pot (see Supplementary Fig. S6 online). Such residues are often observed within archaeological pottery. Consequently, this thin blackened patina residue layer was sampled at least twice for each pot, the first collection was after cook number 50 (sample collection 8), and therefore the first residue is the product of repeated cooking and charring events of the primary recipe. An additional residue was collected after cooking event 51 corresponding to sample collection 9 (following the recipe change). An additional residue sample was collected after cooking event 54 (sample collection 10), for those experiments continued to that point. These residue data are plotted in time sequence with the macro-remains and absorbed lipids in Fig. 2.

The charred food macro-remains and the adhering thin, organic patina carbonized residues were freeze dried, weighed into tin capsules and their *δ*^13^C and *δ*^15^N values determined at the UC Berkeley Center for Stable Isotope Biogeochemistry (CSIB) using a Vario Isocube Elemental Analyzer coupled to an IsoPrime 100 isotope ratio mass spectrometer (IRMS). Stable isotope values for the ingredients are reported in Table 2 and those for charred food macro-remain and carbonized residues are reported in Table 3. The analytical runs were calibrated using NIST SRM 1547 (peach leaves; *δ*^13^C = – 25.9‰; *δ*^15^N =  + 2.1‰) and additional quality control standards were analyzed including IAEA-CH-6 sucrose (*δ*^13^C = – 10.45‰), bovine liver (*δ*^13^C = – 21.6‰; *δ*^15^N =  + 7.8‰), and spirulina (*δ*^13^C = – 32.1‰; *δ*^15^N =  + 10.9‰). CSIB reports long-term isotopic precision data to be ± 0.1 ‰ for *δ*^13^C values and ± 0.2‰ for *δ*^15^N values. A sub-set of samples were analyzed in replicates to assess homogeneity and intra-sample error and are reported as the average values in Table 3 together with standard deviations.

Throughout the cooking year, at least four interior pottery fabric samples were taken for absorbed lipid and compound-specific carbon isotope compositions. The ceramic fabric samples were taken at the beginning of the experiment (after the very first charring event, sample collection 1), mid-way through the experiment (after the 22nd cooking event, sample collection 4), at the end of the experiment when the primary recipe was cooked a final time (after the 50th cook, sample collection 8), and after the recipes were changed (the 51st cook, sample collection 9; and after the 54th cook, sample collection 10, if possible). Ceramic fabric samples were taken from the body of the pot, from the upper limit of where the ingredients reached when they were being cooked (see Supplementary Fig. S6 online). The exterior of the burnished layer (2 mm depth of the pottery fabric as a 1 cm × 0.5 cm rectangle) was removed using a Dremel before the deeper fabric containing the absorbed lipids were collected for analysis. The fabric powder was placed in microcentrifuge tubes, weighed, then freeze dried before lipid analysis. The experimental pottery powder and reference materials were analysed following the procedure outlined in Correa-Ascencio and Evershed^73^. Recovery of cereal lipids followed the procedure outlined in Hammann and Cramp^33^. Briefly, a known amount of internal standard (*n-*tetratriacontane, 20 μL, 1.0 mg mL^−1^ solution) was added to approximately 50 mg of the powder, then the lipids were extracted using 5 mL of 4% sulfuric acid/methanol solution (*δ*^13^C value measured) or using CHCl_3_:CH_3_OH with sonication for recovery of cereal lipids. Total lipid extracts (TLEs) were trimethylsilylated before gas chromatography (GC) in order to determine the lipid concentrations. The samples with detectable organic components were further analysed using GC-mass spectrometry (GC–MS) and GC-combustion-IRMS (GC-C-IRMS) in order to identify the presence and isotopic composition of lipids (full methods are presented in Supplementary Methods online).

Bulk and compound-specific isotope values, and biomarker compositions were determined for the reference (raw ingredients) materials and recipes in order to provide calibration points, for use in assessing the extent of mixing or replacement of lipids within the pottery vessels. Additional compositional analyses were performed using high temperature GC-high resolution MS to provide additional biomarker evidence for the impacts of cooking of different foods on the formation of organic residues.

## Supplementary information

Supplementary Information.

## Data Availability

All data generated or analyzed during this study are included in this published article (and its Supplementary Information files).
