# Supplementary material for: Interpreting ancient food practices: stable isotope and molecular analyses of visible and absorbed residues from a year-long cooking experiment
Source: Sci Rep. 2020 Aug 27;10:13704. doi: 10.1038/s41598-020-70109-8 (PMC7452889; doi:10.1038/s41598-020-70109-8)
Supplement: Supplementary file 1 — Supplementary Information. [file 41598_2020_70109_MOESM1_ESM.pdf]

Supplementary Material

Interpreting ancient food practices: Stable isotope and molecular analyses of visible and absorbed residues from a year-long cooking experiment

Melanie J. Miller, Helen L. Whelton, Jillian A. Swift, Sophia Maline, Simon Hammann, Lucy J.E. Cramp, Alexandra McCleary, Geoffrey Taylor, Kirsten Vacca, Fanya Becks, Richard P. Evershed, Christine A. Hastorf

Supplementary Methods

Acidified methanol extraction

A known amount of internal standard (*n*-tetratriacontane, 20 µL, 1.0 mg mL^{-1} solution) was added to approximately 35-50 mg of powder. The lipids were then esterified and/or transesterified using 5 mL of 4% sulfuric acid/methanol solution ($\delta^{13}C$ measured) and heated for 1 h at 70°C with mixing every 10 min. The supernatant was removed to a clean test-tube and 2 mL of (DCM) extracted double-distilled water added. The remaining potsherd was washed with 5 mL of *n*-hexane and transferred to test-tubes before centrifuging (2500 rpm, 10 min). The *n*-hexane supernatant was then transferred to the sulfuric acid-methanol solution and whirlimixed to extract the lipids before being transferred to a vial. A further 3 × 3 mL of *n*-hexane was added to the H_2SO_4-methanol solution. The *n*-hexane extracts were combined, and the solvent was then removed under a gentle stream of nitrogen in a heating block at 40°C. An aliquot of the extract was derivatised using 20 µL *N,O*-bis(trimethylsilyl)trifluoroacetamide (BSTFA) containing 1% trimethylchlorosilane (TMCS; Sigma Aldrich) prior to analysis by GC, GC-MS and GC-C-IRMS.

Agilent 7820A GC

Analyses of acid extracted FAMEs TLEs were performed using an Agilent 7820A gas chromatograph, using manual injections. The FID used to monitor column effluent was set to 300°C. Trimethylsilylated FAMEs were introduced to the system via on-column injection (1.0

μL). The analytical column was a 50 m × 0.32 mm (Agilent J&W Scientific) fused silica capillary column coated with a 100% dimethylpolysiloxane HP-1 non-polar stationary phase (0.17 μm). The GC temperature programme was set to hold at 50°C for 1 min, followed by a gradient increase to 300 °C 10°C min^{-1}, the oven was then run isothermally for 10 min. Helium was used as the carrier gas set to constant flow of 2.0 mL min^{-1}. Data was acquired using HP Chemstation software (Rev. C.01.07 [27] Agilent Technologies) and eluted peaks were identified by comparison of retention times with those of an external standard, quantification was calculated using a known amount of internal standard introduced during sample preparation.

ThermoScientific ISQ Single Quadrupole GC-MS

GC-MS analyses of trimethylsilylated FAME TLEs aliquots were performed using a ThermoScientific Trace 1300 gas chromatograph couple to an ISQ single quadrupole mass spectrometer. Samples were introduced via a PTV injector set to splitless mode onto a 50 m × 0.32 mm fused silica capillary column coated with a Rtx-1 stationary phase (100% dimethylpolysiloxane, Restek, 0.17 μm) for non-polar analyses. The GC temperature programme for was set to hold at 50°C for 1 min, followed by a gradient increase to 300°C at 10°C min^{-1}, once at 300°C the oven was run isothermally for 10 min. Helium was used as the carrier gas, set to a constant flow of 2 mL min^{-1}. The MS was operated in electron ionisation (EI) mode operating at 70 eV, with a GC transfer line temperature of 300°C and a source temperature of 300°C. The emission current was set to 150 μA and the MS was set to acquire in the range of *m/z* 50-650 at 2 scans s^{-1} in full scan mode. The data acquisition and processing were carried out using XCalibur software, version 3.0. Compounds were identified by comparison with the NIST mass spectra library (version 2.0) or with reference to external sources such as The Lipid Library (www.lipidlibrary.aocs.org)

IsoPrime 100 GC-C-IRMS

Compound-specific carbon stable isotope analyses were performed using an Agilent Industries 7890A gas chromatograph coupled to an IsoPrime 100 mass spectrometer. Samples were introduced via a split/splitless injector in splitless mode onto a 50 m × 0.32 mm fused silica

capillary column coated with a HP-1 stationary phase (100% dimethylpolysiloxane, Agilent, 0.17 µm). The GC oven temperature programme was set to hold at 40°C for 2 min, followed by a gradient increase to 300°C at 10°C min^{-1}, the oven was then run isothermally for 10 min. Helium was used as a carrier gas and maintained at a constant flow of 2 mL min^{-1}. The combustion reactor consisted of a quartz tube filled with copper oxide pellets which was maintained at a temperature of 850°C. Instrument accuracy was determined using an external FAME standard mixture (C_{11}, C_{13}, C_{16}, C_{21} and C_{23}) of known isotopic composition (determined using a two-point calibration using IAEA-CH-7 ($\delta^{13}C$ value = -32.2 ± 0.05‰) and FIRMS phenacetin ($\delta^{13}C$ value = -26.7 ± 0.2‰). Samples were run in duplicate and an average taken (difference between duplicates < 0.5‰). The $\delta^{13}C$ values are the ratios $^{13}C/^{12}C$ and expressed relative to the Vienna Pee Dee Belemnite (VPDB, calibrated against a CO_2 reference gas of known isotopic composition). Instrument error was ± 0.3‰. Data processing was carried out using Ion Vantage software (version 1.5.6.0, IsoPrime).

Cereal biomarker analyses

A known amount of internal standard (*n*-tetratriacontane, 20 µL, 1.0 mg mL^{-1} solution) was added to approximately 35-50 mg of powder. Lipids were then extracted using 2 × 10 mL chloroform/methanol (2:1 *v/v*) with sonication for 20 min. The extract was then centrifuged (10 min, 2500 rpm) and the supernatant transferred to a clean glass vial where the solvent was then removed under nitrogen. The extract was dissolved in 1 mL chloroform/methanol (2:1 *v/v*) with sonication and a 250 µL aliquot filtered through a silica gel column (Silica 60A, particle size 35-70 µm, Fisher Scientific) into a clean glass vial. The solvent was then removed under a gentle stream of nitrogen. Dried sample aliquots were derivatised using 20 µL BSTFA containing 1% trimethylchlorosilane (TMCS; Sigma Aldrich) for 1 h at 70°C. Excess BSTFA was removed under N_2 and the trimethylsilyated total lipid extract (TLE) was diluted with *n*-hexane prior to analysis by HT-GC and GC-MS.

Agilent 7890A GC

All HT-GC analyses of trimethylsilylated TLEs were performed using an Agilent Industries 7890A gas chromatograph, using either manual or autosampler injections. The flame ionisation detector (FID) used to monitor column effluent was set to 350°C. Trimethylsilylated TLEs were introduced to the system via on-column injection (1.0 µL). The analytical column was a 15 m × 0.32 mm (Agilent J&W Scientific) fused silica capillary column coated with a 100% dimethylpolysiloxane DB-1 HT non-polar stationary phase (0.1 µm). The GC temperature programme was set to hold at 50°C for 2 min, followed by a gradient increase to 350°C at 10 °C min^{-1}, the oven was then held isothermally for 10 min. Helium was used as the carrier gas and maintained at a constant flow of 4.26 mL min^{-1}. For the GC analyses of FAMEs, the GC temperature programme was set to hold at 50 °C for 1 min, followed by a gradient increase to 350°C at a 25°C min^{-1}, the oven was then run isothermally for 5 min. Data was acquired using HP Chemstation software (Rev. B.03.02 [341] Agilent Technologies) and eluted peaks were identified by comparison of retention times with those of an external standard. Quantification used a known amount of IS introduced during sample preparation as described above.

Agilent 7890/7200B GC-Q-ToF-MS

HT-GC-MS analyses of TLEs were performed using an Agilent 7890/7200B GC-Q-ToF-MS. Samples (1.0µL) were injected using a 7693 autosampler and a cool on-column inlet (set to follow the oven temperature) onto a 15 m × 0.25 mm x 0.1 µm ZB-5 Inferno column (Phenomenex). The GC temperature programme for was set to hold at 55°C for 2 min, followed by a gradient ramp to 220°C at 10°C min^{-1} and then 350°C at 20 °C min^{-1} before a final isothermal at 350°C for 13 min. Helium was used as the carrier gas set to a constant flow of 1.5 mL min^{-1}. The MS was operated in electron ionisation (EI) mode operating at 70 eV, the GC transfer line, ion source and quadrupole were set to 350°C, 230°C and 150°C, respectively. The MS was set to acquire in the range of *m/z* 50-1050 at 5 scans s^{-1} in Extended Dynamic Range mode.

Mixing model results using IsoSource

IsoSource requires multiple sources for the program to operate (n+1, where n is the number of elements being measured) so for our samples with carbon and nitrogen stable isotope values each condition had 3 sources listed. Since all recipes were only one or two ingredients (sources) we would list those ingredients in replicates for the IsoSource program to function (i.e if the recipe was a mixture of maize and deer then we would list maize twice and deer twice so that the software functioned properly). In cases where three ingredients had been cooked in the pot (so experiments where the recipe swap introduced a new resource), then the three different sources were listed as inputs. In cases where there was only one ingredient used in the primary recipe (such as all maize flour for KV_1 and MM_1) and maize was input as the only source the program of course returned 100% for that resource (we didn't list those results in Supplementary Table S1 because they were always 100%). Therefore, we opted to always use two sources (or three when applicable for cases such as AM and KV) to test the mixing model (see Supplementary Table S2). When we provide the mixing model with an erroneous source that we know was not present, such as wheat for experiments MM_1 and KV_1 (known recipe was 100% maize flour), then the mixing model will allow for a small proportion of the (non-present) resource to contribute to the mixture (even though we know it was not present). For both MM_1 and KV_1 experiments we see that the known 100% maize flour recipe returns an estimate of 95-100% maize and 0-5% wheat. This is both one of the benefits and limitations of mixing models, they are only as good as the source data provided to them. In many cases we want some uncertainty to be expressed, since we know that resources can be consumed in small proportions but not be registered in the final isotopic value (this is true across things like organic residues from pottery, bone collagen, and other materials tested for isotopic data). For archaeologists this is a good feature because it reminds us to think about things that may have been minor contributions to human diet and may be masked through questions of equifinality. All modeling with IsoSource version 1.3.1 was tested at an increment of 5 and tolerance of 1.

Supplementary Figures

Supplementary Figure S1. Partial GC profile of the acid methanol extracted FAME GT #10 with 100% wheat; illustrating the distribution of compounds characteristic of a degraded plant lipids. Key: $FA_{X:Y}$ are free fatty acids of carbon length X and degree of unsaturation Y. IS is the added internal standard (C_{34} *n*-alkane).

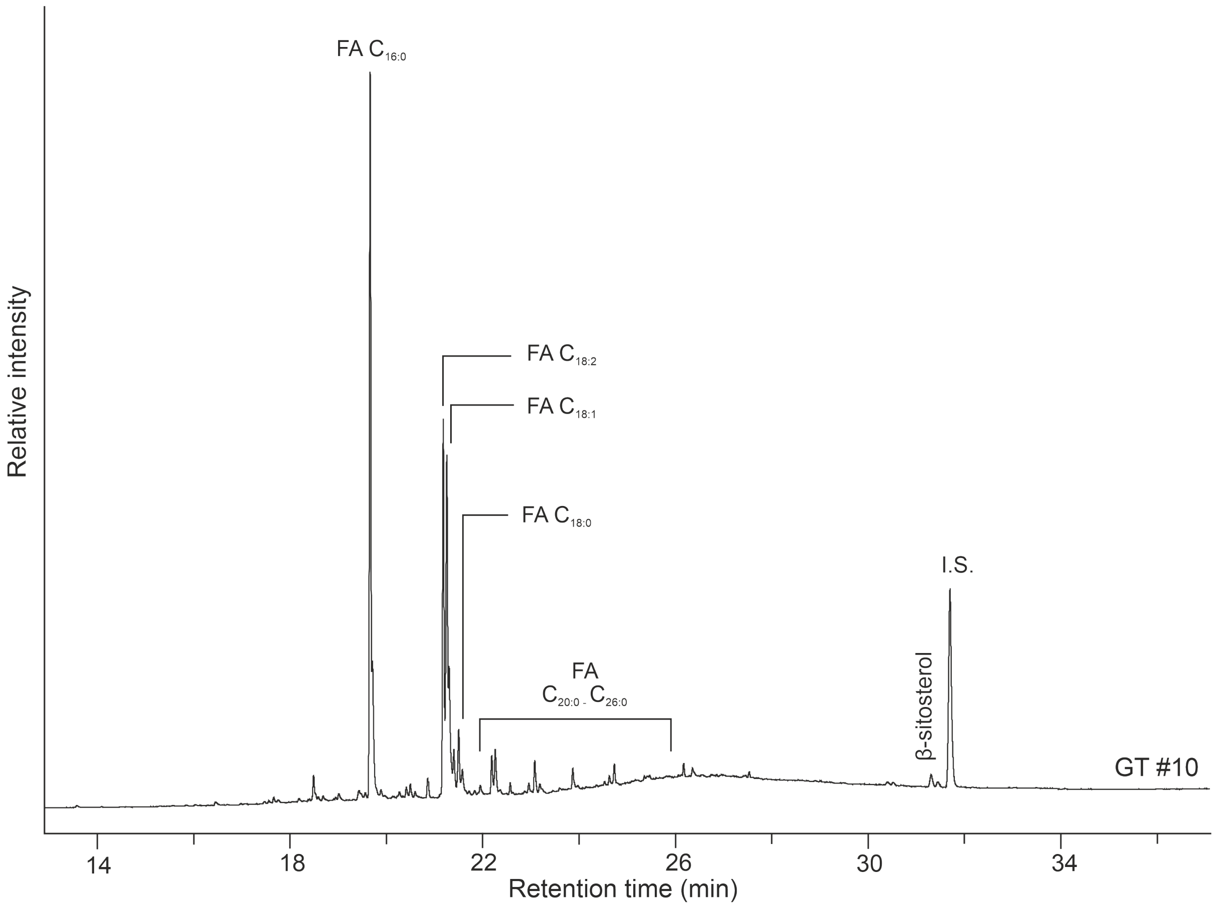

Supplementary Figure S2. Partial GC profile of the acid methanol extracted FAME MM #8 with 100% maize; illustrating the distribution of compounds characteristic of a degraded plant lipids. Key: $FA_{X:Y}$ are free fatty acids of carbon length X and degree of unsaturation Y. IS is the added internal standard (C_{34} *n*-alkane).

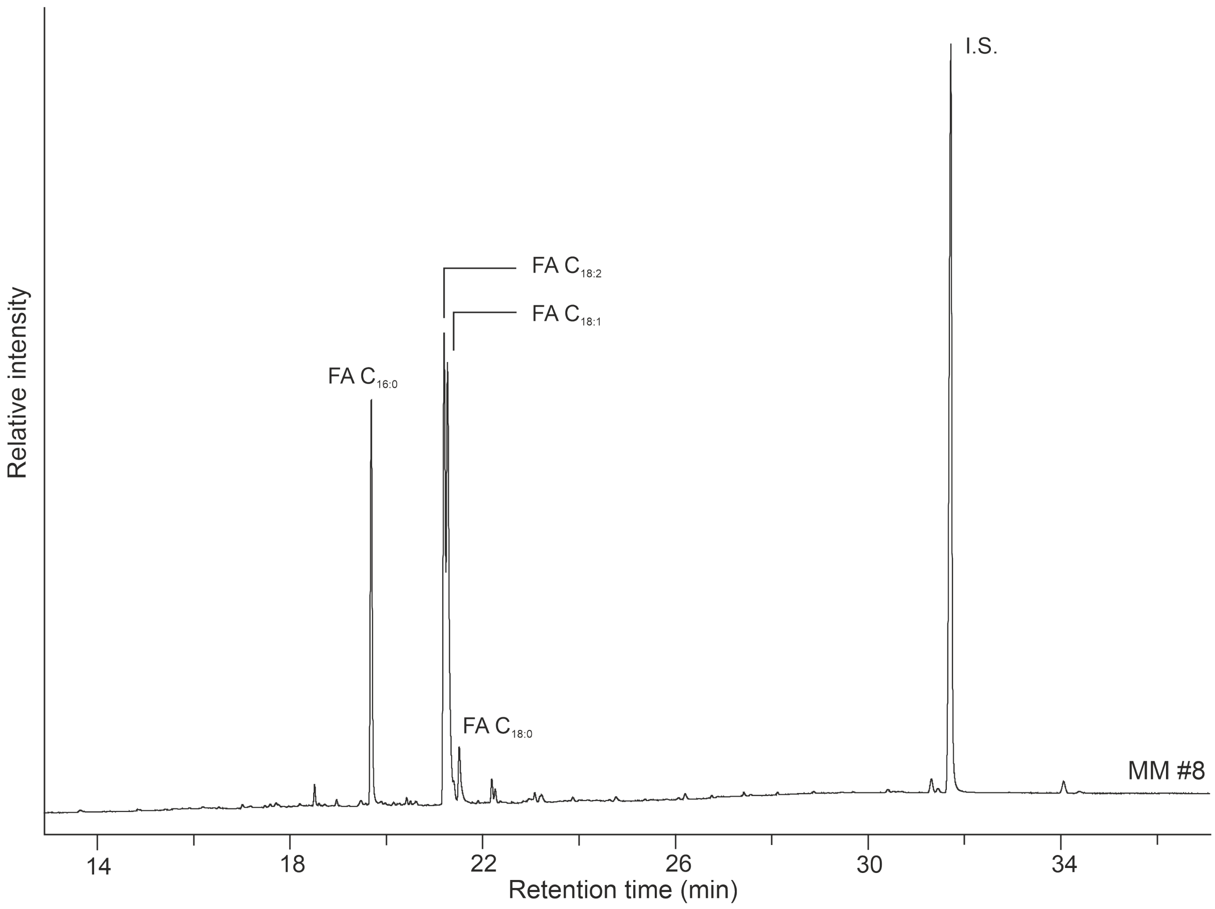

Supplementary Figure S3. Characteristic cereal biomarker lipids from experiments FB #8 and FB #10. Where a) shows the trimethylsilyated sitosterol and absence of sitosterol fatty acid esters in 100% maize displayed using the extracted ion trace of *m/z* 396.3756; b) shows the trimethylsilyated sitosterol and sitosterol fatty acid esters from the 100% wheat displayed using the extracted ion trace of *m/z* 396.3756.

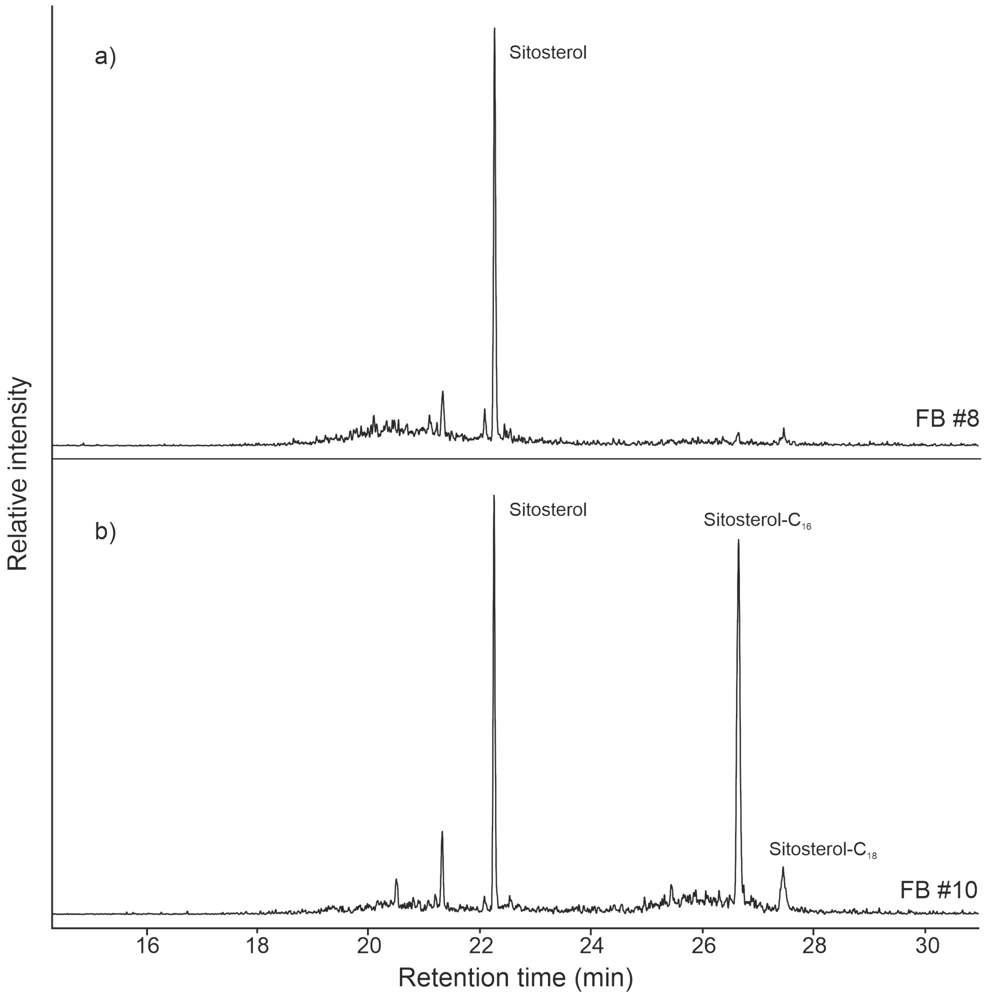

Supplementary Figure S4. Characteristic cereal biomarker lipids from the experiments JS #8 and JS #10. Where a) shows the trimethylsilyated sitosterol and sitosterol fatty acid esters in 25% maize, 75% wheat displayed using the extracted ion trace of *m/z* 396.3756; b) shows the trimethylsilyated sitosterol and sitosterol fatty acid esters in 100% maize displayed using the extracted ion trace of *m/z* 396.3756.

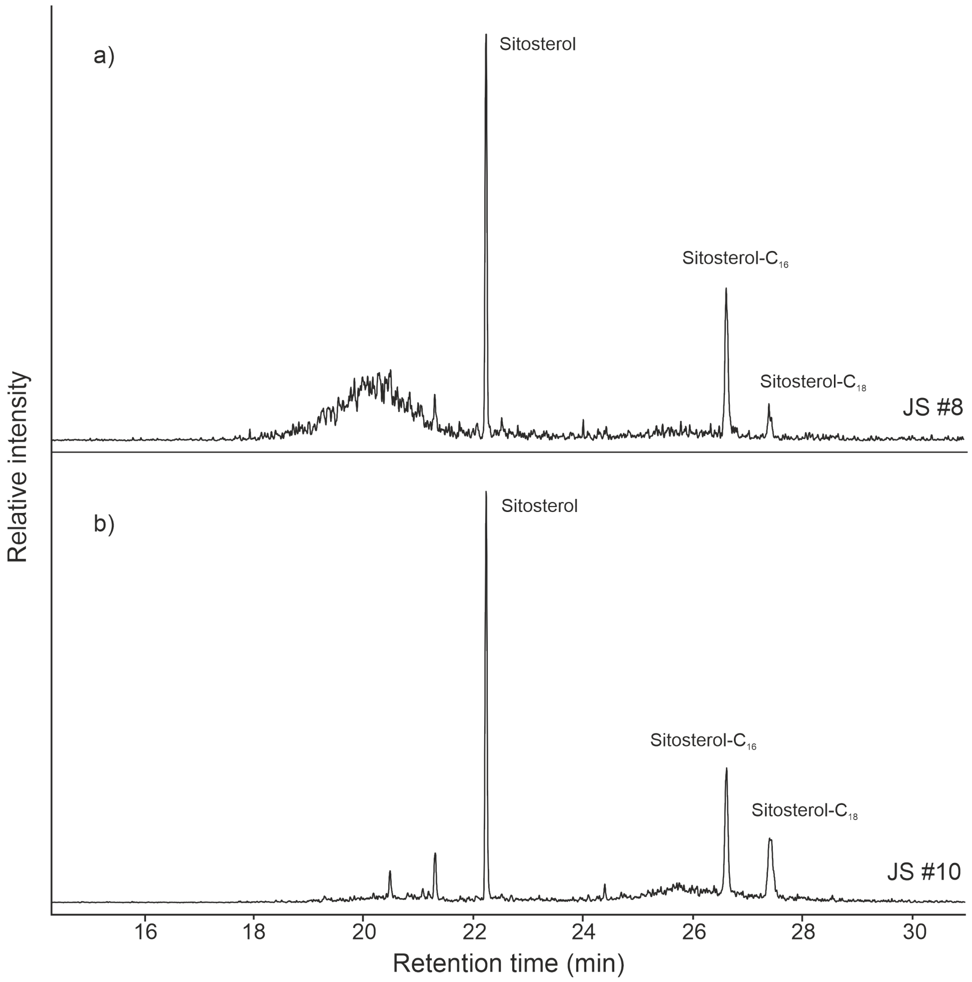

Supplementary Figure S5. Characteristic cereal biomarker lipids from the experiments FB #10 (a) and GT #10 (b) showing the alkylresorcinols present in 100% wheat displayed using the extracted ion trace of *m/z* 268.1315.

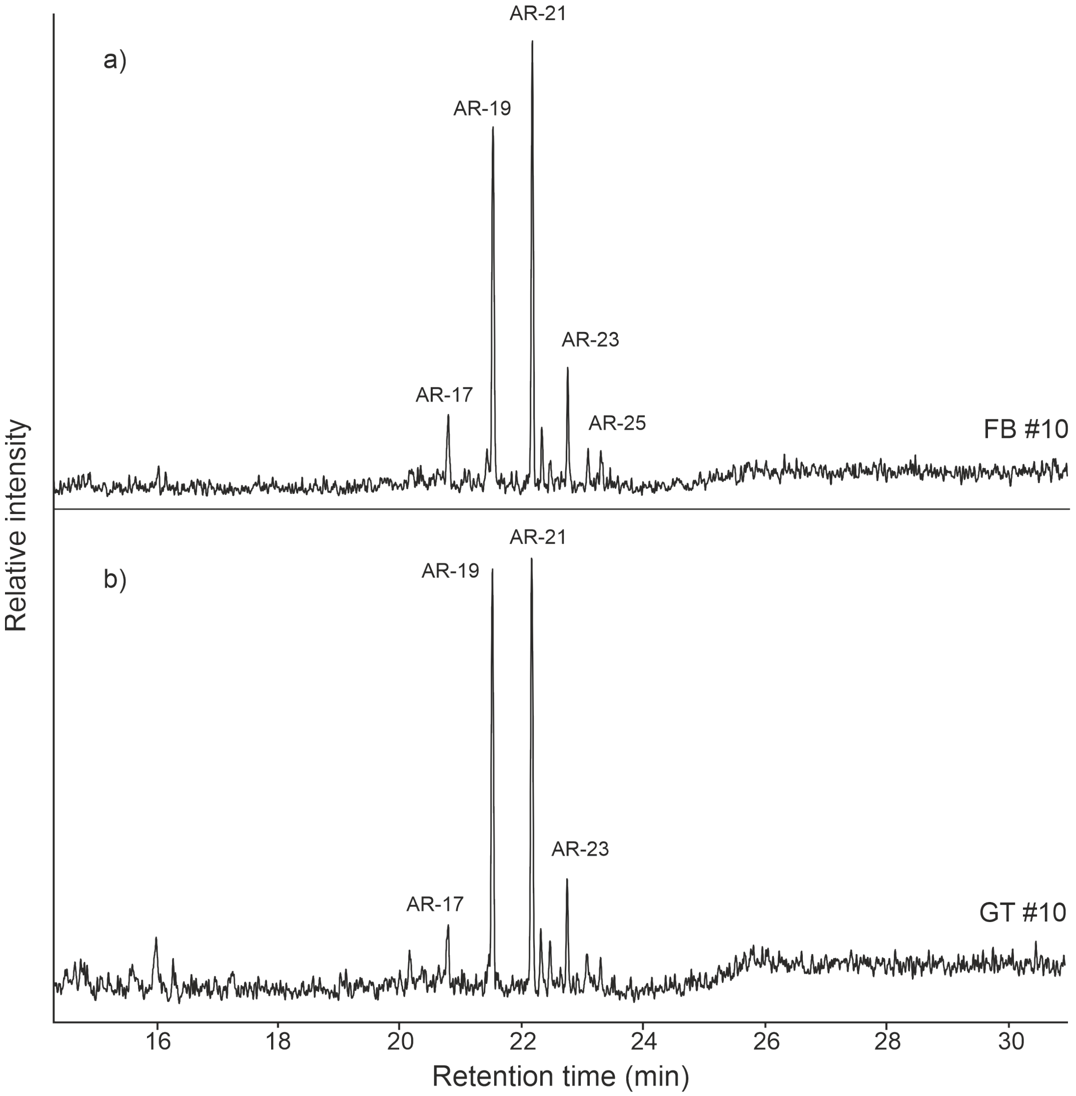

Supplementary Figure S6. Example of the appearance of a cooking pot (experiment JS) at the conclusion of project. The red rectangles indicate where ceramic fabric samples were taken over the course of the experimental data collection period. The yellow circle highlights an area of the organic patina thin-layer residue which accumulated at the bottom of the vessels and was sampled at least twice for each experiment.

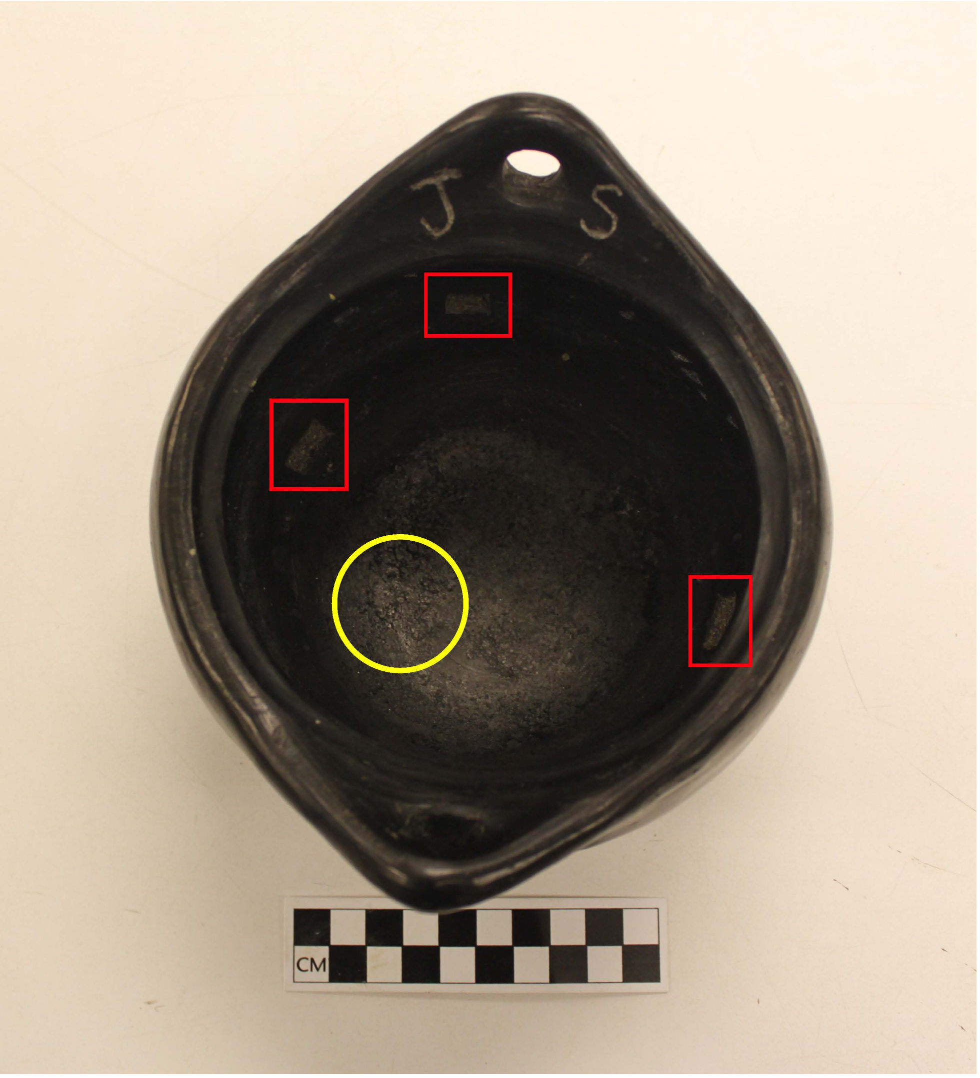

Supplementary Table S1: Mixing model results using IsoSource (v.1.3.1) to estimate percent contribution of various sources to each residue sample. Note all results presented here were analyzed with two sources (even when recipe had only 1 known source/ingredient), and when applicable three sources. Results from experiments FB, GT, KV, MM are presented from sample collection 6 onwards as results were redundant for sample collections 1-8 (all were single ingredient primary recipes).

Chef ID	Sample Collection Number	Sample ID	Recipe	Sample Type	δ13C	δ15N	Percent Hominy	Percent Deer	Percent Wheat
AM	1	AM_1	Hominy & Deer (3:1)	Macro-remains	-15.7	5.3	65-75	25-35	
AM	2	AM_2	Hominy & Deer (3:1)	Macro-remains	-16.0	4.4	65-75	25-35	
AM	3	AM_3	Hominy & Deer (3:1)	Macro-remains	-13.9	5.2	80-85	15-20	
AM	4	AM_4	Hominy & Deer (3:1)	Macro-remains	-12.1	4.7	90-100	0-10	
AM	5	AM_5	Hominy & Deer (3:1)	Macro-remains	-14.5	5.4	75-80	20-25	
AM	6	AM_6	Hominy & Deer (3:1)	Macro-remains	-12.0	4.9	90-100	0-10	
AM	7	AM_7	Hominy & Deer (3:1)	Macro-remains	-11.7	5.0	90-100	0-10	
AM	8	AM_8	Hominy & Deer (3:1)	Macro-remains	-11.7	4.4	90-100	0-10	
AM	8	AM_8r	Hominy & Deer (3:1)	Thin-Layer Residue	-14.6	5.7	75-80	20-25	
AM	9	AM_9	Hominy & Wheat (3:1)	Macro-remains	-15.3	3.8	70-75	0-30	0-30
AM	9	AM_9r	Hominy & Wheat (3:1)	Thin-Layer Residue	-19.6	4.5	40-50	20-60	0-30

Chef ID	Sample Collection Number	Sample ID	Recipe	Sample Type	δ13C	δ15N	Percent Maize	Percent Wheat
CH	1	CH_1	Maize Flour & Wheat (1:1)	Macro-remains	-19.0	3.0	45-55	45-55
CH	2	CH_2	Maize Flour & Wheat (1:1)	Macro-remains	-18.4	2.9	50-55	45-50

CH	3	CH_3	Maize Flour & Wheat (1:1)	Macro-remains	-19.3	3.2	45-50	50-55
CH	4	CH_4	Maize Flour & Wheat (1:1)	Macro-remains	-18.3	3.0	50-55	45-50
CH	5	CH_5	Maize Flour & Wheat (1:1)	Macro-remains	-21.6	2.3	25-35	65-75
CH	6	CH_6	Maize Flour & Wheat (1:1)	Macro-remains	-17.9	2.8	50-60	40-50
CH	7	CH_7	Maize Flour & Wheat (1:1)	Macro-remains	-22.1	2.5	25-30	70-75
CH	8	CH_8	Maize Flour & Wheat (1:1)	Macro-remains	-18.1	2.9	50-60	40-50
CH	8	CH_8r	Maize Flour & Wheat (1:1)	Thin-Layer Residue	-19.6	3.3	40-50	50-60
CH	9	CH_9	Maize Flour	Macro-remains	-13.6	4.0	80-90	10-20
CH	9	CH_9r	Maize Flour	Thin-Layer Residue	-17.7	3.7	55-60	40-45
CH	10	CH_10	Maize Flour	Macro-remains	-12.5	4.0	85-95	5-15
CH	10	CH_10r	Maize Flour	Thin-Layer Residue	-15.9	3.7	65-75	25-35

**Chef ID**	**Sample Collection Number**	**Sample ID**	**Recipe**	**Sample Type**	**δ13C**	**δ15N**	**Percent Maize**	**Percent Wheat**
JS	1	JS_1	Wheat & Maize Flour (3:1)	Macro-remains	-15.2	3.2	70-80	20-30
JS	2	JS_2	Wheat & Maize Flour (3:1)	Macro-remains	-22.9	2.2	20-25	75-80
JS	3	JS_3	Wheat & Maize Flour (3:1)	Macro-remains	-22.6	2.3	20-30	70-80
JS	4	JS_4	Wheat & Maize Flour (3:1)	Macro-remains	-23.9	2.5	10-20	80-90
JS	5	JS_5	Wheat & Maize Flour (3:1)	Macro-remains	-23.9	2.3	10-20	80-90

JS	6	JS_6	Wheat & Maize Flour (3:1)	Macro-remains	-23.3	2.8	20-25	75-80
JS	7	JS_7	Wheat & Maize Flour (3:1)	Macro-remains	-23.1	2.5	20-25	75-80
JS	8	JS_8	Wheat & Maize Flour (3:1)	Macro-remains	-23.5	2.2	15-25	75-85
JS	8	JS_8r	Wheat & Maize Flour (3:1)	Thin-Layer Residue	-22.8	2.7	20-30	70-80
JS	9	JS_9	Maize Flour	Macro-remains	-11.2	4.3	95-100	0-5
JS	9	JS_9r	Maize Flour	Thin-Layer Residue	-17.7	3.4	55-60	40-45
JS	10	JS_10	Maize Flour	Macro-remains	-11.1	4.2	95-100	0-5
JS	10	JS_10r	Maize Flour	Thin-Layer Residue	-17.7	3.3	55-60	40-45

**Chef ID**	**Sample Collection Number**	**Sample ID**	**Recipe**	**Sample Type**	**δ13C**	**δ15N**	**Percent Maize**	**Percent Wheat**
FB	6	FB_6	Whole Maize	Macro-remains	-11.1	5.3	100	0
FB	7	FB_7	Whole Maize	Macro-remains	-11.2	5.1	95-100	0-5
FB	8	FB_8	Whole Maize	Macro-remains	-11.1	4.2	95-100	0-5
FB	8	FB_8r	Whole Maize	Thin-Layer Residue	-10.5	5.5	95-100	0-5
FB	9	FB_9	Wheat Flour	Macro-remains	-26.4	2.2	0-5	95-100
FB	9	FB_9r	Wheat Flour	Thin-Layer Residue	-16.5	4.4	65-75	25-35
FB	10	FB_10	Wheat Flour	Macro-remains	-26.3	1.7	0-5	95-100
FB	10	FB_10r	Wheat Flour	Thin-Layer Residue	-20.4	3.6	30-45	55-70

**Chef ID**	**Sample Collection Number**	**Sample ID**	**Recipe**	**Sample Type**	**δ13C**	**δ15N**	**Percent Hominy**	**Percent Wheat**

Chef ID	Sample Collection Number	Sample ID	Recipe	Sample Type	δ13C	δ15N	Percent Maize	Percent Wheat	Percent Deer
GT	6	GT_6	Hominy	Macro-remains	-11.1	4.9	95-100	0-5	
GT	7	GT_7	Hominy	Macro-remains	-11.2	5.0	95-100	0-5	
GT	8	GT_8	Hominy	Macro-remains	-11.2	5.1	95-100	0-5	
GT	8	GT_8r	Hominy	Thin-Layer Residue	-11.1	4.7	95-100	0-5	
GT	9	GT_9	Wheat Flour	Macro-remains	-26.1	2.0	0-5	95-100	
GT	9	GT_9r	Wheat Flour	Thin-Layer Residue	-22.9	2.7	20-25	75-80	
GT	10	GT_10	Wheat Flour	Macro-remains	-26.3	2.1	0-5	95-100	
GT	10	GT_10r	Wheat Flour	Thin-Layer Residue	-24.9	2.5	5-15	85-95	
**Chef ID**	**Sample Collection Number**	**Sample ID**	**Recipe**	**Sample Type**	**δ13C**	**δ15N**	**Percent Maize**	**Percent Wheat**	**Percent Deer**
KV	6	KV_6	Maize Flour	Macro-remains	-11.0	4.4	95-100	0-5	
KV	7	KV_7	Maize Flour	Macro-remains	-11.1	4.4	95-100	0-5	
KV	8	KV_8	Maize Flour	Macro-remains	-11.0	4.5	95-100	0-5	
KV	8	KV_8r	Maize Flour	Thin-Layer Residue	-11.1	4.3	95-100	0-5	
KV	9	KV_9	Wheat & Deer (3:1)	Macro-remains	-26.4	4.9	0-5	0-25	70-100
KV	9	KV_9r	Wheat & Deer (3:1)	Thin-Layer Residue	-22.3	3.4	25-35	5-60	10-65
**Chef ID**	**Sample Collection Number**	**Sample ID**	**Recipe**	**Sample Type**	**δ13C**	**δ15N**	**Percent Maize**	**Percent Wheat**	**Percent Deer**
MM	6	MM_6	Maize Flour	Macro-remains	-11.0	4.4	95-100	0-5	
MM	7	MM_7	Maize Flour	Macro-remains	-11.0	4.3	95-100	0-5	
MM	8	MM_8	Maize Flour	Macro-remains	-11.0	4.3	95-100	0-5	
MM	8	MM_8r	Maize Flour	Thin-Layer Residue	-11.1	4.6	95-100	0-5	

MM	9	MM_9	Maize Flour & Deer (3:1)	Macro-remains	-11.5	4.3	95-100	0-5
MM	9	MM_9r	Maize Flour & Deer (3:1)	Thin-Layer Residue	-11.8	4.6	90-100	0-10

Supplementary Table S2: Compound-specific isotope data for reference materials

**Reference material**	**$\delta\ ^{13}C_{16:0}$ (‰)**
100% ground maize	-17.7
100% ground wheat	-33.4
50% maize, 50% wheat	-26.5
75% wheat, 25% maize	-28.7
Whole maize kernel	-19.4
Whole wheat kernel	-34.2
Hominy	-23.6
Deer meat	-33.5
